# Applications of artificial intelligence in traditional Chinese medicine tongue diagnosis: a bibliometric review of research trends and hotspots, 2015–2026

**DOI:** 10.3389/fphys.2026.1895902

**Published:** 2026-10-05

**Authors:** Yilin Li, Yang Yang, Shaoqin Lin, Hengxu Wang, Liyuan Yan, Mingxiang Zheng, Lei Xiang, Xiangjiang Tang

**Affiliations:** 1 Department of Traditional Chinese Medicine, Guangzhou Cadre and Talent Health Management Center, Guangzhou, China; 2 College of Nursing, Changsha Medical University, Changsha, China; 3 Imaging Department, Clinical Research Center for Reproduction and Genetics in Hunan Province, Reproductive & Genetic Hospital of CITIC-Xiangya, Changsha, China

**Keywords:** artificial intelligence, bibliometric analysis, CiteSpace, tongue diagnosis, traditional Chinese medicine, VOSviewer

## Abstract

**Objectives:**

This study aimed to characterize the development landscape of artificial intelligence (AI)-assisted tongue diagnosis in Traditional Chinese Medicine (TCM) by comparing publication trends, collaboration patterns, research hotspots, and emerging topics between CNKI-indexed and WoSCC-indexed literature.

**Methods:**

Literature published between January 2015 and August 2026 was retrieved from the Web of Science Core Collection (WoSCC) and China National Knowledge Infrastructure (CNKI). Publication trends were summarized descriptively, and CiteSpace and VOSviewer were used for collaboration-network, keyword co-occurrence, clustering, timeline, burst-detection, and journal citation analyses. CNKI-indexed and WoSCC-indexed records were analysed separately because of differences in database coverage, indexing characteristics, metadata structures, and publication practices.

**Results:**

A total of 482 studies were included, comprising 295 WoSCC-indexed and 187 CNKI-indexed records. Publication output showed an overall upward trend in both datasets over the study period. The CNKI keyword clustering network showed high clustering quality (modularity Q = 0.7929; weighted mean silhouette S = 0.9413), as did the WoSCC network (Q = 0.8000; S = 0.9239). In CNKI, the strongest keyword bursts included “deep learning” (strength = 2.95), “image segmentation” (2.69), and “transfer learning” (2.61); in WoSCC, prominent bursts included “artificial intelligence” (2.13), “image colour analysis” (2.10), and “machine learning” (1.86). CNKI-indexed literature showed prominent themes involving tongue-image objectification, segmentation, and TCM-oriented diagnostic concepts, whereas WoSCC-indexed literature showed more differentiated themes involving AI architectures, tongue-feature analysis, computational modelling, and disease-related applications. Highly cited publications in both datasets further reflected the importance of tongue-image extraction and segmentation and the subsequent expansion toward disease-oriented modelling. Across both datasets, the temporal patterns suggested a shift in research attention from conventional image processing and feature extraction toward machine learning, deep learning, and more integrated AI-based approaches.

**Conclusions:**

The two datasets showed complementary but database-specific thematic structures in AI-assisted tongue diagnosis. Although the literature indicates a shift toward more advanced and disease-oriented AI applications, these bibliometric patterns do not establish the scientific or medical validity, diagnostic accuracy, or clinical readiness of AI-assisted tongue analysis. Further standardization, multicentre external validation, and clinically interpretable and reproducible research are needed before broader clinical application.

## 1 Introduction

Traditional Chinese Medicine (TCM) is a medical system with a long history of clinical practice (Cyranoski, 2018). Among its diagnostic approaches, tongue diagnosis represents an important component of TCM clinical assessment. According to TCM theory, changes in the tongue and tongue coating are used to evaluate physiological and pathological conditions (Han et al., 2016). Tongue characteristics, including colour, morphological features, and coating characteristics, have therefore been investigated for disease-related assessment (Han et al., 2016). Because tongue examination is non-invasive, convenient, and inexpensive, it has attracted increasing attention as a potential approach for objective health assessment (Ma et al., 2019).

However, conventional tongue diagnosis remains largely dependent on practitioners’ experience and subjective interpretation, which may affect diagnostic consistency (Segawa et al., 2023). Variations in image acquisition conditions, preprocessing procedures, and feature representation may affect the performance and robustness of automated tongue analysis (Zhang et al., 2017; Ma et al., 2019; Xu et al., 2020). Therefore, establishing objective, standardized, and quantitative approaches for tongue assessment has become an important direction in the modernization of TCM diagnosis (Xu et al., 2020; Segawa et al., 2023).

The development of computerized tongue diagnosis has provided new opportunities to overcome the limitations of traditional subjective evaluation. Early studies mainly relied on digital image processing techniques and manually designed features, including colour and texture characteristics, combined with conventional machine-learning algorithms (Zhang et al., 2017; Xu et al., 2020). Zhang et al. developed a diabetes diagnostic model based on standardized tongue images using support vector machine (SVM), demonstrating the feasibility of extracting colour and texture features from tongue images for disease classification (Zhang et al., 2017).

With the rapid development of artificial intelligence (AI), particularly computer vision and deep learning technologies, tongue image analysis has gradually shifted from handcrafted feature engineering toward automatic feature learning (Li et al., 2018; Ma et al., 2019; Xu et al., 2020). Li et al. proposed a tooth-marked tongue recognition method based on multiple-instance learning and CNN features, showing that CNN-derived features could be used to recognize complex morphological variations of tooth-marked tongues (Li et al., 2018). Ma et al. further developed a deep-learning-based framework for tongue constitution recognition, incorporating tongue detection, calibration, feature extraction, and constitution classification, thereby reducing reliance on manually designed features (Ma et al., 2019). More recently, AI-assisted tongue diagnosis has evolved toward integrated intelligent analysis frameworks. Xu et al. proposed a multi-task joint learning model that simultaneously performed tongue segmentation and classification using deep neural networks, demonstrating that shared feature representations between related tasks could improve tongue characterization performance (Xu et al., 2020).

Despite substantial technological progress, several challenges remain before AI-assisted tongue diagnosis can achieve widespread clinical application. Variations in image acquisition conditions, preprocessing procedures, and feature representation may affect the robustness and generalizability of automated tongue analysis (Zhang et al., 2017; Ma et al., 2019; Xu et al., 2020). More broadly, medical AI research continues to face challenges related to bias, transparency, generalizability, rigorous clinical validation, and reproducibility (Topol, 2019; Liu et al., 2020; Vasey et al., 2022). High algorithmic performance obtained during model development does not necessarily translate directly into clinical benefit, highlighting the importance of rigorous validation and transparent reporting before clinical implementation (Liu et al., 2020; Vasey et al., 2022).

The rapid growth of AI-assisted tongue diagnosis research has resulted in an increasingly complex and multidisciplinary research landscape (Tian et al., 2023; Liu et al., 2026). Many individual studies have focused on specific algorithms, model optimization, or particular clinical applications (Zhang et al., 2017; Li et al., 2018; Ma et al., 2019; Xu et al., 2020). Although these investigations provide important technical insights, individual studies cannot fully characterize the overall evolution of the field, including publication trends, collaboration networks, knowledge structures, and changing research priorities. Bibliometric analysis provides a quantitative framework for mapping scientific development through publication, citation, collaboration, and thematic relationships (Chen, 2006; van Eck and Waltman, 2010).

Previous bibliometric studies have investigated AI applications in TCM diagnosis. Tian et al. conducted a scientometric analysis of AI research involving the four diagnostic methods of TCM and identified tongue-image classification and diagnosis, particularly approaches involving deep neural networks, as important research hotspots (Tian et al., 2023). More recently, Liu et al. conducted a bibliometric analysis specifically focusing on AI-assisted tongue diagnosis; its bibliometric component included English-language articles and reviews retrieved from the Web of Science Core Collection (WoSCC) and identified research themes involving AI and deep-learning architectures, image processing and segmentation, TCM-specific applications, and disease-specific applications (Liu et al., 2026).

However, previous bibliometric analyses were primarily based on WoSCC and did not directly compare Chinese-language CNKI literature with internationally indexed WoSCC publications (Tian et al., 2023; Liu et al., 2026). Chinese-language publications indexed in CNKI represent an important component of TCM-related research and may reflect distinct research priorities, developmental trajectories, and knowledge dissemination patterns. Therefore, a comparative bibliometric analysis of CNKI and WoSCC covering the period from 2015 to 2026 may provide complementary perspectives on the evolution, knowledge structure, and emerging directions of AI-assisted tongue diagnosis. Accordingly, this bibliometric review aimed to compare CNKI- and WoSCC-indexed literature on artificial intelligence in TCM tongue diagnosis from 2015 to August 2026, with a focus on publication trends, collaboration patterns, highly cited publications, research hotspots, thematic evolution, and emerging research directions.

## 2 Methods

### 2.1 Study design and data sources

This bibliometric study examined research on artificial intelligence (AI)-assisted tongue diagnosis in traditional Chinese medicine (TCM) using records retrieved from the China National Knowledge Infrastructure (CNKI) and the Web of Science Core Collection (WoSCC). The study period covered publications from January 1, 2015 to the respective final search dates in August 2026. This period was selected to capture the recent development of AI-assisted tongue diagnosis, particularly the increasing application of machine learning, deep learning, computer vision, and related computational methods over the past decade.

CNKI and WoSCC were selected to represent two complementary publication environments. CNKI provides extensive coverage of Chinese-language academic literature and is particularly relevant to TCM research and its domestic applications, whereas WoSCC provides structured bibliographic and citation metadata for internationally indexed literature. However, the two databases differ substantially in indexing scope, document-selection policies, citation practices, and metadata structures. Therefore, CNKI and WoSCC records were cleaned, analysed, and interpreted separately. Cross-database comparisons were intended to characterize differences in publication patterns, collaboration structures, and research themes between CNKI-indexed Chinese-language literature and WoSCC-indexed English-language literature rather than to provide an exhaustive representation of all Chinese and international research on AI-assisted tongue diagnosis.

The reporting completeness of this bibliometric review was assessed against the BIBLIO guideline for reporting bibliometric reviews of the biomedical literature (Montazeri et al., 2023), and the completed checklist is provided in **Supplementary Table S6**.

### 2.2 Search strategy

The search strategy was developed around three conceptual domains: AI and computational methods, tongue diagnosis, and TCM-related concepts. Database-specific strategies were adopted to balance retrieval sensitivity and topic specificity. For CNKI, the three conceptual domains were combined in the final search. For WoSCC, a broader two-concept search was used as the primary high-sensitivity retrieval strategy, while an additional three-concept search incorporating TCM-related terminology was conducted to assess the effect of increasing topic specificity.

For CNKI, the final search combined subject terms related to AI/computational methods, tongue diagnosis, and TCM. AI-related terms covered major computational approaches, including artificial intelligence, machine learning, deep learning, neural networks, computer vision, image processing and recognition, pattern recognition, feature extraction, support vector machines, random forests, Bayesian methods, data mining, generative adversarial networks, and Transformer-based methods. Tongue-related terms covered tongue images, tongue diagnosis, tongue colour, tongue coating, tongue body, tongue morphology, texture, image analysis, recognition, classification, segmentation, and objective tongue diagnosis. TCM-related terms included traditional Chinese medicine, TCM diagnosis, syndrome differentiation, syndrome/pattern, constitution, the four diagnostic methods, and inspection diagnosis.

The initial CNKI search retrieved 1,524 records. Restricting the publication period to January 1, 2015 through August 14, 2026 yielded 1,208 records, and restricting the document type to journal articles yielded 297 records for eligibility screening. Only Chinese-language publications were eligible for inclusion; English-language records retrieved from CNKI were excluded during screening. The final CNKI search was conducted on August 14, 2026.

For WoSCC, two related search strategies were conducted to balance retrieval sensitivity and topic specificity. Both searches were restricted to English-language Articles and Review Articles published between January 1, 2015 and August 15, 2026.

The primary high-sensitivity search combined AI/computational terms with tongue-diagnosis terms without requiring TCM-related terminology and retrieved 3,716 records after application of the predefined publication-period, language, and document-type restrictions.

A second, more specific search added a third concept block containing TCM-related terms and retrieved 703 records under the same restrictions. The addition of the TCM concept block substantially reduced the number of retrieved records. Because relevant studies on AI-assisted tongue diagnosis may not explicitly mention TCM-related terminology in their titles, abstracts, or keywords, restricting the corpus to this three-concept search alone could reduce retrieval sensitivity. The 703 records retrieved by the more specific search were contained within the broader 3,716-record corpus. Therefore, the broader high-sensitivity corpus of 3,716 unique records was retained for systematic eligibility screening, while topic relevance to TCM tongue diagnosis was subsequently determined according to the predefined eligibility criteria.

To quantitatively assess the impact of using the more specific strategy alone, the final 295 eligible WoSCC publications were cross-matched against the 703 records retrieved by the TCM-specific search. Of the 295 included publications, 221 (74.9%) were captured by the TCM-specific search, whereas 74 (25.1%) were retrieved only by the broader search and would therefore have been missed if the more specific strategy had been used alone. Given that the more specific strategy would have omitted 74 eligible publications, the broader high-sensitivity search was retained as the basis for eligibility screening. Both WoSCC searches were conducted on August 15, 2026.

The complete database-specific search strings, field tags, language and document-type restrictions, search dates, and numbers of retrieved records are provided in **Supplementary Table S1**.

### 2.3 Eligibility criteria

Studies were included if they met the following criteria: (1) application of AI or related computational methods; (2) tongue images or quantitatively derived tongue features constituted a substantive component of the study; (3) tongue-related information was used for TCM tongue diagnosis, tongue-feature recognition or classification, syndrome/pattern differentiation, constitution assessment, disease-related assessment, or other clinically relevant TCM-related applications; and (4) the publication met the database-specific language and document-type requirements (Chinese-language journal articles for CNKI and English-language Articles or Review Articles for WoSCC).

Studies were excluded if they: (1) focused on the anatomical or functional tongue without relevance to TCM tongue diagnosis, such as speech, swallowing, tongue movement, or articulation; (2) focused on oral or dental diseases, oral microbiology, tongue tumours, or other oral pathology without a TCM tongue-diagnosis component; (3) mentioned the tongue only incidentally or did not apply AI/computational methods to substantive tongue-image or tongue-feature analysis; or (4) were duplicate records or non-eligible publication types, including conference abstracts/proceedings, patents, newspapers, errata, and retracted publications.

Conference proceedings were excluded because this study focused on peer-reviewed journal literature concerning the medical application of AI in TCM tongue diagnosis. This restriction also improved consistency in document type and bibliographic metadata across CNKI and WoSCC. However, because emerging AI methods may initially be disseminated through conference proceedings, their exclusion may have resulted in underrepresentation of some recent technological developments; this potential limitation was considered when interpreting temporal trends.

### 2.4 Literature screening and study selection

All candidate records were screened according to the predefined eligibility criteria. Two researchers independently screened all candidate records by reviewing titles, abstracts, author keywords, and other available bibliographic information. Full texts were reviewed when the available information was insufficient to determine eligibility. Disagreements between the two reviewers were resolved through discussion, with consultation of a third researcher when necessary.

For CNKI, 297 journal articles entered eligibility screening after application of the publication-period and document-type restrictions. Following title and abstract screening, 101 records were excluded and 196 were retained. Of these, 9 records for which eligibility remained uncertain underwent full-text assessment and were excluded, leaving 187 studies in the final CNKI dataset.

For WoSCC, all 3,716 records retrieved through the broader high-sensitivity search underwent independent eligibility screening by two reviewers. Following title and abstract screening, 3,316 records were excluded, leaving 400 records. Of these, 105 records for which eligibility could not be determined reliably from the title and abstract alone underwent full-text assessment and were subsequently excluded according to the predefined eligibility criteria. The remaining 295 studies constituted the final WoSCC dataset.

Because CNKI and WoSCC differ substantially in bibliographic field structures and indexing conventions, potential cross-database duplicates were assessed manually rather than relying solely on automated matching. Article titles and authors were initially compared to identify potentially overlapping records. Potential matches were subsequently verified using abstracts, journal/source information, publication year, and DOI when available. No duplicate records were identified between the final CNKI and WoSCC datasets.

A total of 187 CNKI records and 295 WoSCC records were included in the final analysis, with the two datasets analysed separately. The complete literature identification and screening process is presented in a study-selection flow diagram (**Figure 1**).

**Figure 1:**
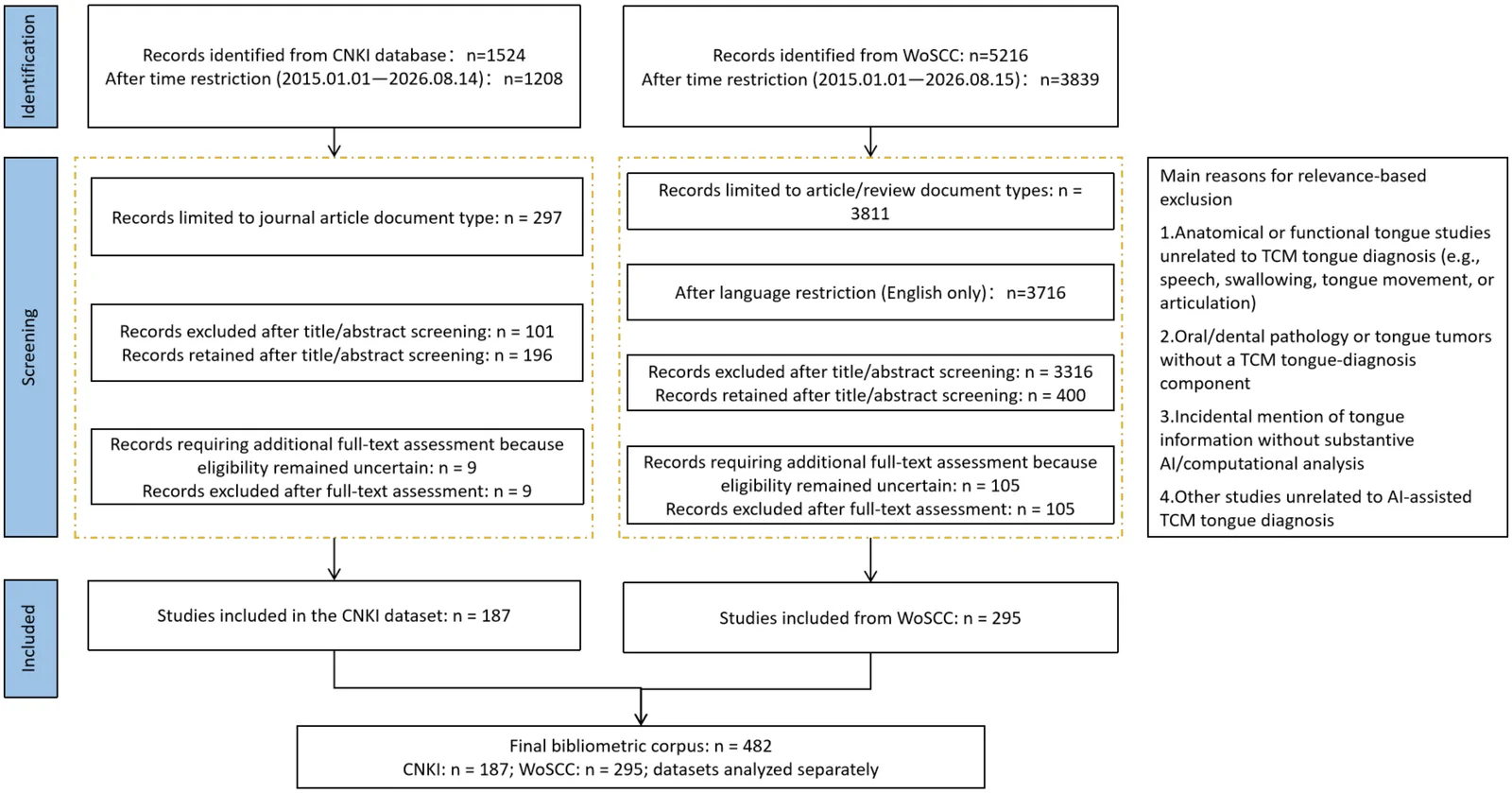
Flow diagram of the literature search and study selection process for the CNKI and WoSCC datasets.

### 2.5 Data cleaning and standardization

Bibliographic data from CNKI and WoSCC were processed separately before analysis. Author names were reviewed for obvious spelling, hyphenation, and transliteration variants. Clearly identifiable variants referring to the same author were merged when supported by the available bibliographic information; for example, “Xu, Jiatuo” and “Xu, Jia-tuo,” “Tu, Liping” and “Tu, Li-ping,” and “Hu, Xiaojuan” and “Hu, Xiao-juan” were standardised as the same authors. Institutional fields were reviewed for obvious inconsistencies in naming and indexing, but no systematic manual merging of institutional nodes was performed. Keyword processing was performed separately for the two datasets. For the WoSCC dataset, English keyword nodes were standardised conservatively by merging abbreviations and their full forms, singular/plural variants, spelling variants, and clearly equivalent expressions referring to the same concept. Terms with potentially distinct technical, methodological, or clinical meanings were retained separately to avoid over-standardisation. For the country/region collaboration analysis, the “TAIWAN” node was merged into “PEOPLES R CHINA” for geographic aggregation. For cross-dataset presentation, Chinese keywords were translated into English using standardised TCM and biomedical terminology, while the original Chinese terms were retained during data verification.

For descriptive characterisation of the included corpus, application orientation, AI/computational approach, tongue-diagnosis task, and disease/application area were coded from titles and author keywords, with abstracts reviewed when necessary. Multiple classifications were permitted for AI/computational approach, tongue-diagnosis task, and disease/application area.

The cleaned CNKI and WoSCC datasets were subsequently analysed separately. Given differences in database size, indexing structure, metadata characteristics, and publication practices, network characteristics were first interpreted within each dataset, and cross-dataset comparisons focused primarily on relative patterns and thematic structures rather than direct comparisons of absolute frequencies.

### 2.6 Data analysis

Considering the heterogeneity of the CNKI and WoSCC datasets in terms of database size, indexing structure, metadata characteristics, and publication practices, CiteSpace and VOSviewer were selected as complementary bibliometric tools. CiteSpace (version 6.4.R1) was used for time-sliced network analyses, including author, institutional, and country/region collaboration networks, keyword co-occurrence analysis, keyword clustering and timeline visualization, citation burst detection, and betweenness-centrality analysis, owing to its strengths in revealing the temporal evolution and structural characteristics of scientific knowledge networks (Chen, 2004). VOSviewer (version 1.6.21) was used primarily for journal citation analysis and complementary network visualization because of its strengths in bibliometric similarity mapping, network layout, and label readability (van Eck and Waltman, 2010). The two tools were therefore used for complementary analytical purposes rather than for formal cross-software validation. Microsoft Excel was used for descriptive summaries of publication characteristics, including annual publication trends, journal-related metrics, publication year, country/region distribution, and citation rankings.

Exponential trendlines were fitted descriptively using complete annual publication counts from 2015 to 2025. Because 2026 represented a partial publication year, it was displayed descriptively but excluded from trend fitting; the fitted equations were not used for forecasting.

In CiteSpace analyses, the time span was set from 2015 to 2026 with a 1-year time slice. The g-index was used as the node-selection criterion, with a scaling factor of k = 25 for network construction in both datasets. The node types analysed included authors, institutions, countries/regions, and keywords. For text processing, all available term sources were selected, including Title, Abstract, Author Keywords (DE), and Keywords Plus (ID). For keyword co-occurrence analysis, the networks were generated without pruning to retain the overall co-occurrence structure. For keyword clustering and timeline analyses, Pathfinder pruning and pruning of the merged network were applied to reduce redundant links and improve the interpretability of the network structure. Consequently, the number of links in the clustering networks could differ from that in the corresponding unpruned co-occurrence networks, even when the same set of keyword nodes was retained. Cluster labels were generated using the log-likelihood ratio (LLR) method. Keyword bursts were detected using CiteSpace, with a minimum burst duration of 1 year, and burst strength and duration were recorded to characterise temporary increases in research attention. Unless otherwise specified, all other CiteSpace parameters were retained at their default settings. For VOSviewer journal citation analysis, full counting and association-strength normalisation were applied.

Because this study was designed to characterize bibliometric and thematic patterns rather than to synthesize clinical effectiveness, diagnostic accuracy, or intervention effects, no formal methodological quality or risk-of-bias assessment of individual publications was performed.

The analyses included publication trend analysis, author, institutional, and country/region collaboration analysis, journal citation analysis, keyword co-occurrence analysis, keyword clustering and timeline analysis, keyword burst detection, and citation ranking of highly cited publications.

## 3 Results

To clarify the comparative structure of the findings, the Results are presented in two broad parts: structural characteristics of the literature, including publication, collaboration, journal, and citation patterns; and thematic and temporal development, examined through keyword co-occurrence, clustering, timeline, and burst analyses.

### 3.1 Number of publications

Annual publication counts were used to describe the temporal distribution of research output and were not interpreted as direct indicators of scientific quality or clinical impact. The annual publication trends of CNKI-indexed Chinese-language literature and WoSCC-indexed English-language literature are presented in **Figure 2**.

**Figure 2:**
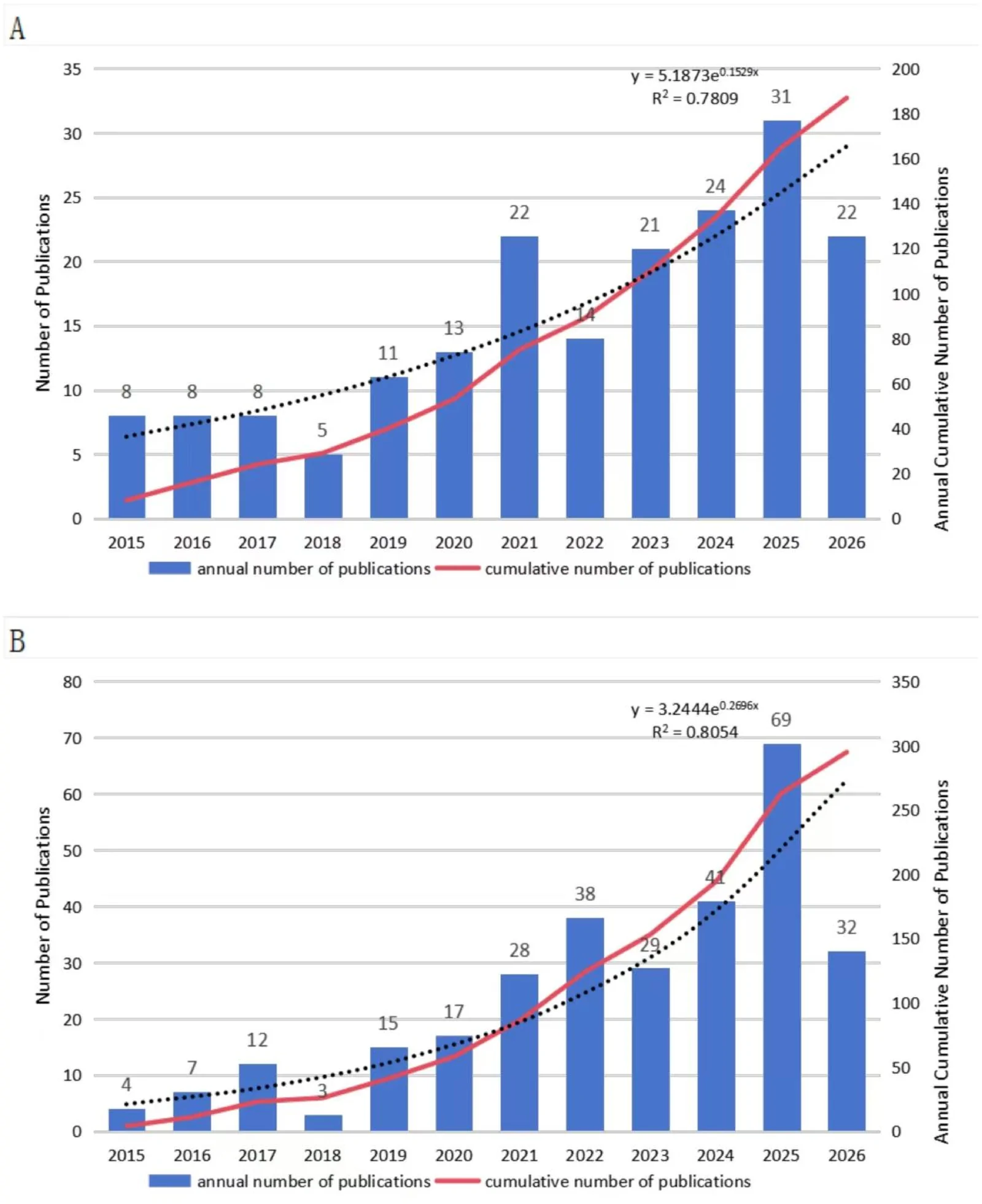
Annual and cumulative publication trends in the CNKI and WoSCC datasets from 2015 to 2026. **(A)** CNKI dataset; **(B)** WoSCC dataset. Bars represent the annual number of publications, solid lines represent the cumulative number of publications, and dotted lines represent exponential trendlines fitted using complete annual data from 2015 to 2025. Publication counts for 2026 represent records available up to the respective retrieval dates in August 2026 and were excluded from exponential trend fitting.

Overall, publication output showed an increasing trend in both datasets. In the CNKI dataset (**Figure 2A**), 8 publications were identified in 2015, and annual output reached its highest level in 2025, with 31 publications. In the WoSCC dataset (**Figure 2B**), the number of publications increased from 4 in 2015 to a peak of 69 in 2025. By the respective retrieval dates in August 2026, 22 CNKI publications and 32 WoSCC publications had been identified. Because these 2026 counts represent partial-year data, they were retained for descriptive presentation but excluded from trend fitting.

To characterize the temporal publication trajectories, exponential trendlines were fitted using complete annual publication data from 2015 to 2025. The CNKI dataset yielded an exponential fitting equation of *y* = 5.1873e^0.1529^*x* (R^2^ = 0.7809), whereas the WoSCC dataset yielded *y* = 3.2444e^0.2696^*x* (R^2^ = 0.8054) (**Figure 2**). These R^2^ values indicate the goodness of fit of the exponential trendlines within the observed complete-year period and were not used to predict future publication output.

Overall, both datasets demonstrated an increasing publication trend during 2015–2026. Publication output increased more rapidly after approximately 2020, and the WoSCC dataset showed a higher annual publication volume than the CNKI dataset in recent years.

### 3.2 Descriptive characteristics of the included literature

The descriptive characteristics of the 482 included publications are summarized in **Supplementary Table S2**. The corpus comprised 187 CNKI-indexed Chinese-language publications and 295 WoSCC-indexed English-language publications. Most records were original journal articles or articles (478/482, 99.2%), while four WoSCC records (0.8%) were indexed as review articles.

In terms of application orientation, general tongue diagnosis and objectification represented the largest category (282/482, 58.5%), followed by disease-specific applications (135/482, 28.0%), TCM syndrome or constitution assessment (47/482, 9.8%), and general health assessment or prediction (18/482, 3.7%). Disease-specific applications were proportionally more frequent in the WoSCC dataset than in the CNKI dataset (32.9% vs. 20.3%).

Regarding computational approaches, deep learning and convolutional neural networks were the most frequently represented methods (233/482, 48.3%), followed by conventional image processing or handcrafted-feature approaches (128/482, 26.6%) and traditional machine-learning methods (81/482, 16.8%). Transformer-, attention-, or large-language-model-related approaches accounted for 12.4%, while multimodal or feature-fusion approaches accounted for 11.0%. The most common tongue-analysis tasks were tongue-phenotype recognition or classification (41.1%), feature extraction or quantitative analysis (40.2%), and disease diagnosis, prediction, or risk assessment (31.7%). Because computational approaches, tongue-analysis tasks, and disease/application areas were coded using non-mutually exclusive categories, individual publications could contribute to more than one category.

### 3.3 Collaborative author network analysis

Author productivity and collaboration networks were analysed separately for the CNKI and WoSCC datasets using CiteSpace 6.4.R1, with the top 10 most productive authors presented in **Table 1**. Core authors were identified according to Price’s law (Zong, 2016), using the following formula:


$$\text{N}=0.749{\left(\text{ηmax}\right)}^{1/2}$$

**Table 1:** Leading authors by publication output in the CNKI and WoSCC datasets.

Rank	CNKI author	Publications, n	WoSCC author	Publications, n
1	Zhang, Xinfeng	9	Xu, Jiatuo	26
2	Xu, Jiatuo	9	Tu, Liping	23
3	Hu, Guangqin	6	Jiang, Tao	17
4	Dong, Changwu	6	Hu, Xiaojuan	17
5	Ren, Jian	6	Cui, Ji	15
6	Zhuo,Li	6	Wen, Guihua	12
7	Tu, Liping	5	Huang, Jingbin	10
8	Xu, Ying	5	Chiang, John Y	9
9	Hu, Xiaojuan	5	Yao, Xinghua	8
10	Li, Fufeng	5	Shi, Yulin	8

Authors were ranked by publication count within each dataset. Where ties occurred at the cutoff, only the first 10 entries in the source ranking were retained for presentation. CNKI, China National Knowledge Infrastructure; WoSCC, Web of Science Core Collection.

Where ηmax represents the number of publications by the most productive author in each dataset. Authors whose publication counts exceeded the calculated threshold were considered core authors.

The CNKI dataset comprised 187 publications, and the author collaboration network contained 279 nodes and 460 links, with a network density of 0.0119. Zhang Xinfeng and Xu Jiatuo were the most productive authors, with 9 publications each, followed by Hu Guangqin, Dong Changwu, Ren Jian, and Zhuo Li, with 6 publications each (**Table 1**). With (ηmax=9), the Price’s law threshold was (N = 2.25); therefore, authors with at least 3 publications were classified as core authors. The collaboration network contained multiple relatively distinct collaborative groups involving Xu Jiatuo, Zhang Xinfeng, Hu Guangqin, Dong Changwu, Tu Liping, and other authors (**Figure 3A**).

**Figure 3:**
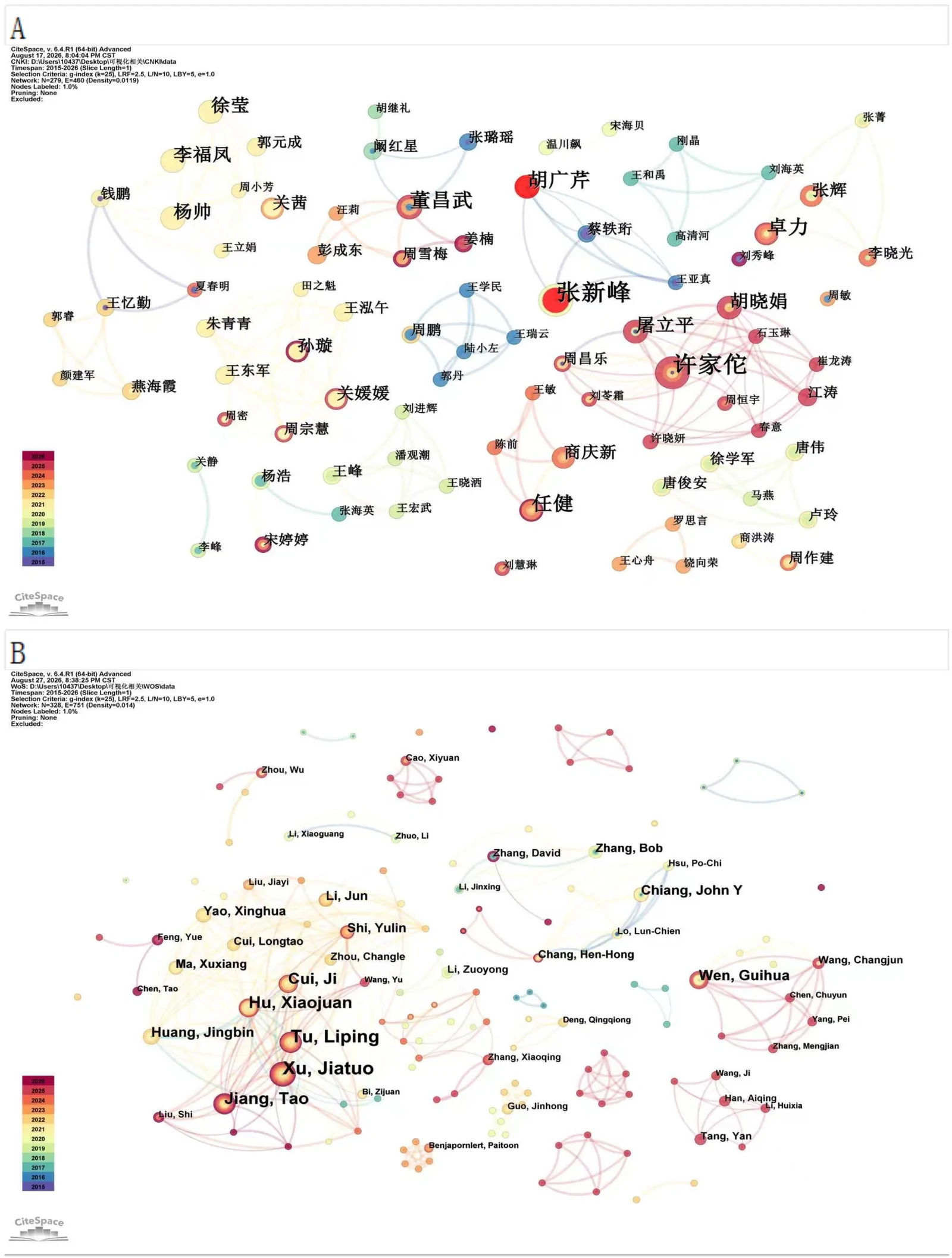
Author collaboration networks in the CNKI- and WoSCC-indexed literature. **(A)** CNKI-indexed literature; **(B)** WoSCC-indexed literature. Node size represents publication frequency, links indicate coauthorship, and colours indicate the temporal distribution of publications. Purple rings indicate nodes with betweenness centrality ≥0.10.

The WoSCC dataset comprised 295 publications, and the author collaboration network contained 328 nodes and 751 links, with a network density of 0.014. Xu Jiatuo was the most productive author, with 26 publications, followed by Tu Liping (23), Jiang Tao and Hu Xiaojuan (17 each), Cui Ji (15), and Wen Guihua (12) (**Table 1**). With ηmax = 26, the corresponding Price’s law threshold was N = 3.82; therefore, authors with at least 4 publications were classified as core authors. The author collaboration network showed a prominent interconnected group involving Xu Jiatuo, Tu Liping, Jiang Tao, Hu Xiaojuan, Cui Ji, Huang Jingbin, Shi Yulin, and other authors, alongside several smaller collaborative groups (**Figure 3B**).

### 3.4 Institutional collaboration network analysis

Institutional productivity and collaboration networks were analysed separately for the CNKI and WoSCC datasets using CiteSpace 6.4.R1, with the top 10 most productive institutions presented in **Table 2**. The CNKI institutional collaboration network contained 189 nodes and 179 links, with a network density of 0.0101, whereas the WoSCC network contained 177 nodes and 346 links, with a density of 0.0222 (**Figure 4**).

**Table 2:** Leading institutions by publication output in the CNKI and WoSCC datasets.

Rank	CNKI institution	Publications, n	WoSCC institution	Publications, n
1	Shanghai University of Traditional Chinese Medicine	8	Shanghai University of Traditional Chinese Medicine	37
2	School of Traditional Chinese Medicine, Shanghai University of Traditional Chinese Medicine	8	Beijing University of Chinese Medicine	24
3	School of Basic Medical Sciences, Shanghai University of Traditional Chinese Medicine	7	Guangzhou University of Chinese Medicine	16
4	Faculty of Information Technology, Beijing University of Technology	7	Xiamen University	15
5	School of Medical Information Engineering, Anhui University of Chinese Medicine	5	Chinese Academy of Sciences	14
6	Affiliated Hospital of Nanjing University of Chinese Medicine	4	South China University of Technology	12
7	Teaching Experimental Center, Shanghai University of Traditional Chinese Medicine	4	China Academy of Chinese Medical Sciences	11
8	School of Artificial Intelligence and Information Technology, Nanjing University of Chinese Medicine	4	Chengdu University of Traditional Chinese Medicine	10
9	Longhua Hospital Affiliated to Shanghai University of Traditional Chinese Medicine	3	China Medical University Taiwan	9
10	Shanghai Innovation Center of Traditional Chinese Medicine Health Service, Shanghai University of Traditional Chinese Medicine	3	Beijing University of Technology	9

Institutions were ranked by publication count within each database. Where ties occurred at the cutoff, only the first 10 entries in the source ranking were retained for presentation. CNKI, China National Knowledge Infrastructure; WoSCC, Web of Science Core Collection.

**Figure 4:**
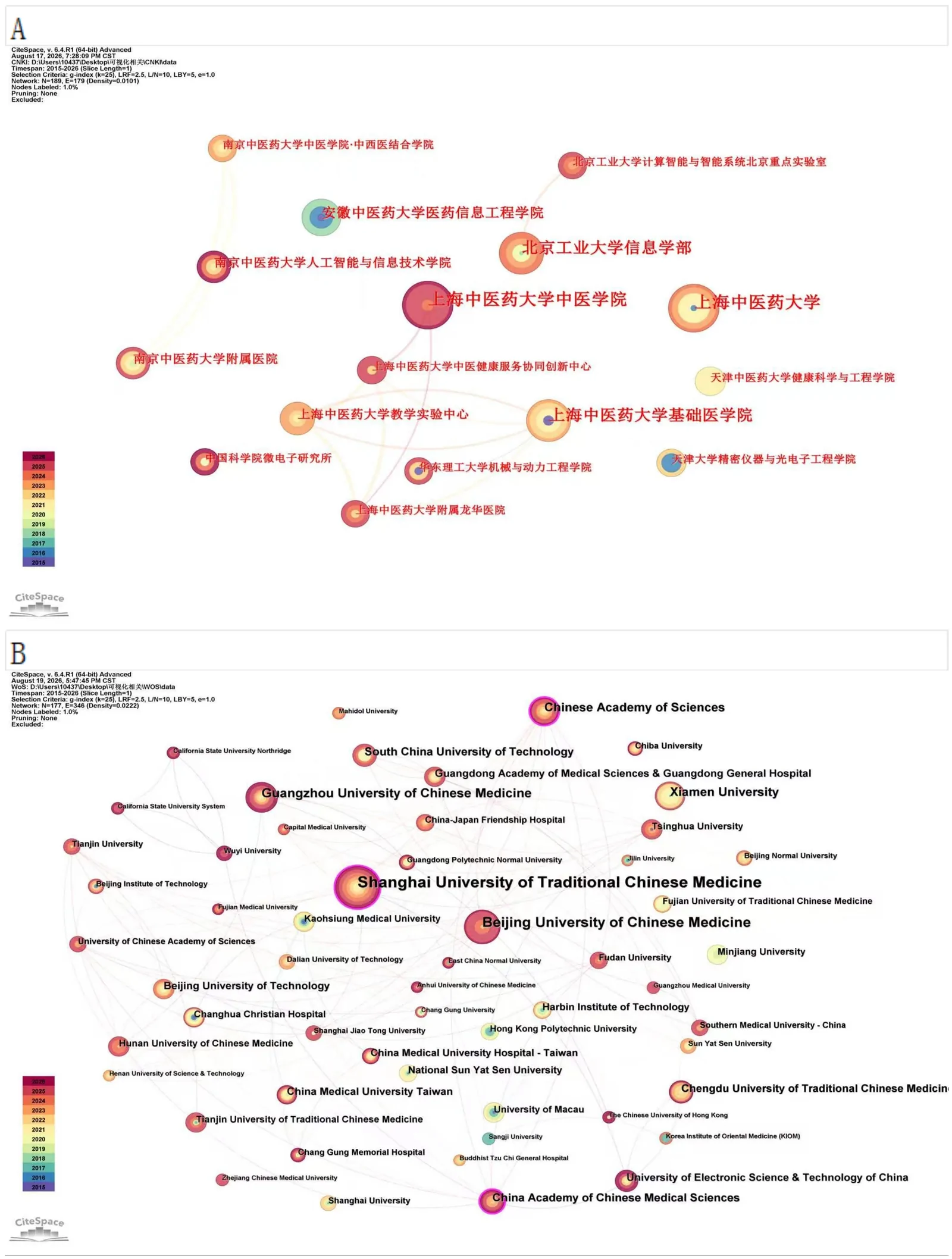
Institutional collaboration networks in the CNKI- and WoSCC-indexed literature. **(A)** CNKI-indexed literature; **(B)** WoSCC-indexed literature. Node size represents publication frequency, links indicate interinstitutional collaboration, and colours indicate the temporal distribution of publications. Purple rings indicate nodes with betweenness centrality ≥0.10.

In the CNKI dataset, Shanghai University of Traditional Chinese Medicine and its School of Traditional Chinese Medicine ranked jointly first, with 8 publications each, followed by the School of Basic Medical Sciences of Shanghai University of Traditional Chinese Medicine and the Faculty of Information Technology, Beijing University of Technology, with 7 publications each. The School of Medical Information Engineering of Anhui University of Chinese Medicine ranked next with 5 publications (**Table 2**). The institutional collaboration network was relatively sparse, with several institutions or institutional groups showing limited interconnections (**Figure 4A**).

In the WoSCC dataset, Shanghai University of Traditional Chinese Medicine was the most productive institution, with 37 publications, followed by Beijing University of Chinese Medicine (24), Guangzhou University of Chinese Medicine (16), Xiamen University (15), and the Chinese Academy of Sciences (14) (**Table 2**). The WoSCC network contained more interinstitutional links and had a higher network density than the CNKI network (0.0222 vs. 0.0101), showing a more interconnected institutional collaboration structure (**Figure 4B**).

### 3.5 Country collaboration network analysis

Country-level collaboration in the WoSCC dataset was analysed using CiteSpace 6.4.R1 (**Figure 5**). The country collaboration network contained 24 nodes and 30 links, with a network density of 0.1087. Node size represents publication frequency, links between nodes indicate international collaboration, and betweenness centrality reflects the structural importance of a country within the collaboration network. Countries with a betweenness centrality ≥0.10 were considered to occupy relatively important bridging positions in the network (Freeman, 1977).

**Figure 5:**
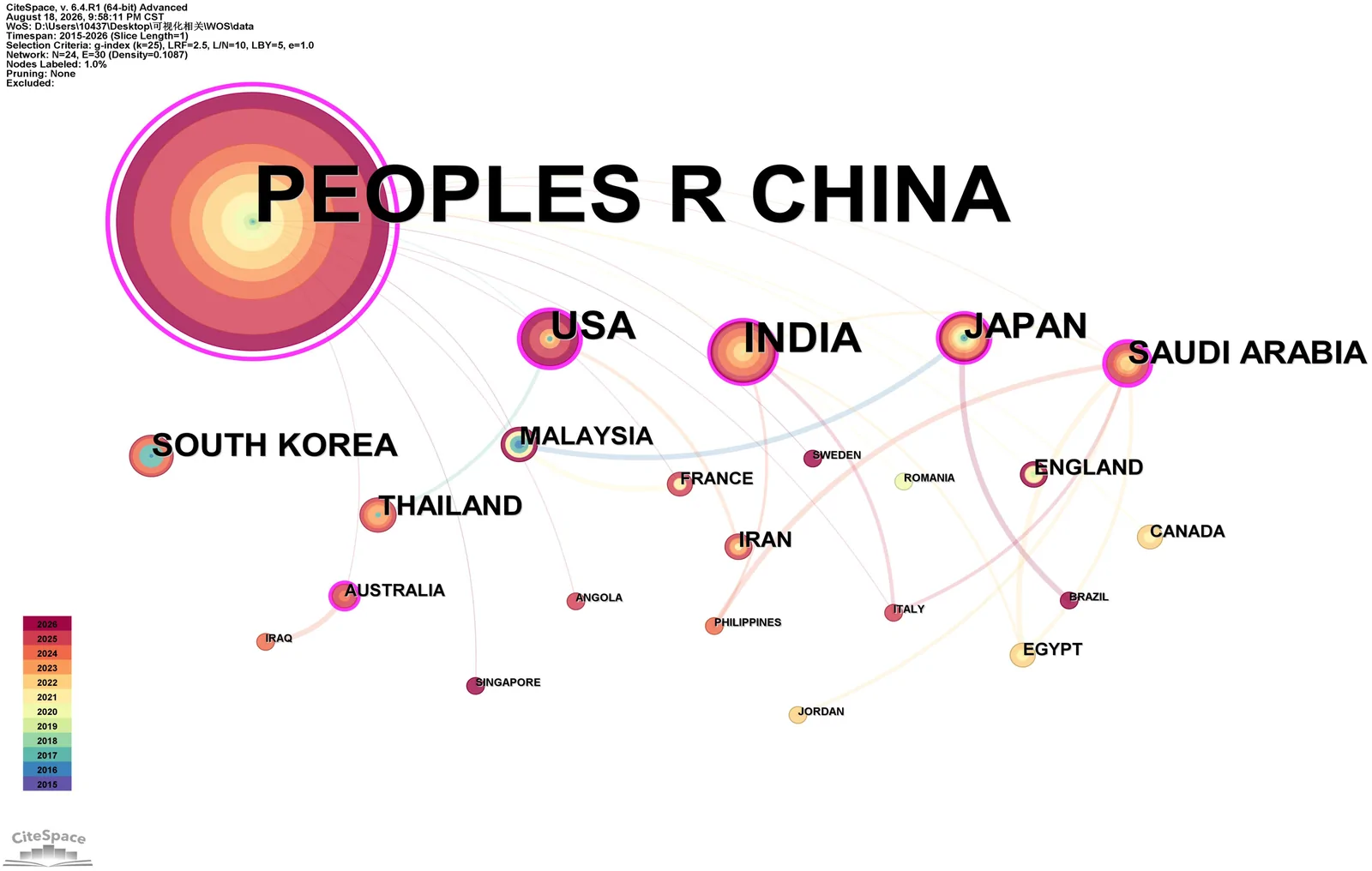
Country collaboration network in the WoSCC-indexed literature. Node size represents publication frequency, links indicate international collaboration, and colours indicate the temporal distribution of publications. Purple rings indicate countries with betweenness centrality ≥0.10.

China was the largest contributor, with 252 publications, followed by India (14), the United States (12), Japan (9), Saudi Arabia (7), and South Korea (7). China also had the highest normalized betweenness centrality (0.81), followed by the United States (0.17) and Saudi Arabia (0.16). These were the only countries with normalized betweenness centrality ≥0.10. Japan and Australia each had a centrality of 0.09, while India had a centrality of 0.08. Overall, China occupied the most prominent position in the WoSCC country collaboration network in terms of both publication output and structural bridging importance (**Figure 5**).

### 3.6 Analysis of publishing journals and cited journals

The distribution of journals publishing AI-assisted tongue diagnosis studies was analysed using VOSviewer. The top ten journals with the highest number of publications are presented in **Table 3**. Among the included studies, IEEE Access published the largest number of articles (n=17), followed by Frontiers in Physiology (n=14) and Scientific Reports (n=12). Other productive journals included Biomedical Signal Processing and Control (n=11), Evidence-Based Complementary and Alternative Medicine (n=10), and Digital Health (n=10).

**Table 3:** Top 10 journals by number of publications in the WoSCC dataset.

Rank	Journal	Publications, n	Country/region	JCR quartile	IF
1	IEEE Access	17	UNITED STATES	Q2	3.4
2	Frontiers in Physiology	14	SWITZERLAND	Q1	4.3
3	Scientific Reports	12	ENGLAND	Q1	4.9
4	Biomedical Signal Processing and Control	11	ENGLAND	Q2	5.7
5	Evidence-Based Complementary and Alternative Medicine	10	ENGLAND	Q3	/
6	Digital Health	10	ENGLAND	Q1	3.9
7	Expert Systems with Applications	7	UNITED STATES	Q1	9.4
8	Applied Sciences	7	SWITZERLAND	Q2	2.9
9	Technology and Health Care	7	NETHERLANDS	Q4	1.3
10	IEEE Journal of Biomedical and Health Informatics	6	UNITED STATES	Q1	7.7

Journal impact factors and JCR quartiles were based on the 2026 Journal Citation Reports. WoSCC, Web of Science Core Collection; IF, impact factor; JCR, Journal Citation Reports.

Several journals with relatively high academic impact were identified. Among the top-ranked journals, Expert Systems with Applications (IF = 9.4) and IEEE Journal of Biomedical and Health Informatics (IF = 7.7) showed relatively high impact factors. These journals mainly cover biomedical engineering, artificial intelligence, medical informatics, and computational medicine, reflecting the interdisciplinary characteristics of AI-assisted tongue diagnosis research.

Journal citation analysis identified the most frequently cited journals within the reference network of AI-assisted tongue diagnosis research. The three most frequently cited journals were *IEEE Access* (213 citations), *IEEE Journal of Biomedical and Health Informatics* (197 citations), and *Evidence-Based Complementary and Alternative Medicine* (143 citations). Other frequently cited journals included *Computers in Biology and Medicine* (141 citations) and *BioMed Research International* (131 citations). These citation frequencies represent the number of times journals were cited within the cocitation network and should not be interpreted as direct indicators of journal quality or research impact (**Figure 6**).

**Figure 6:**
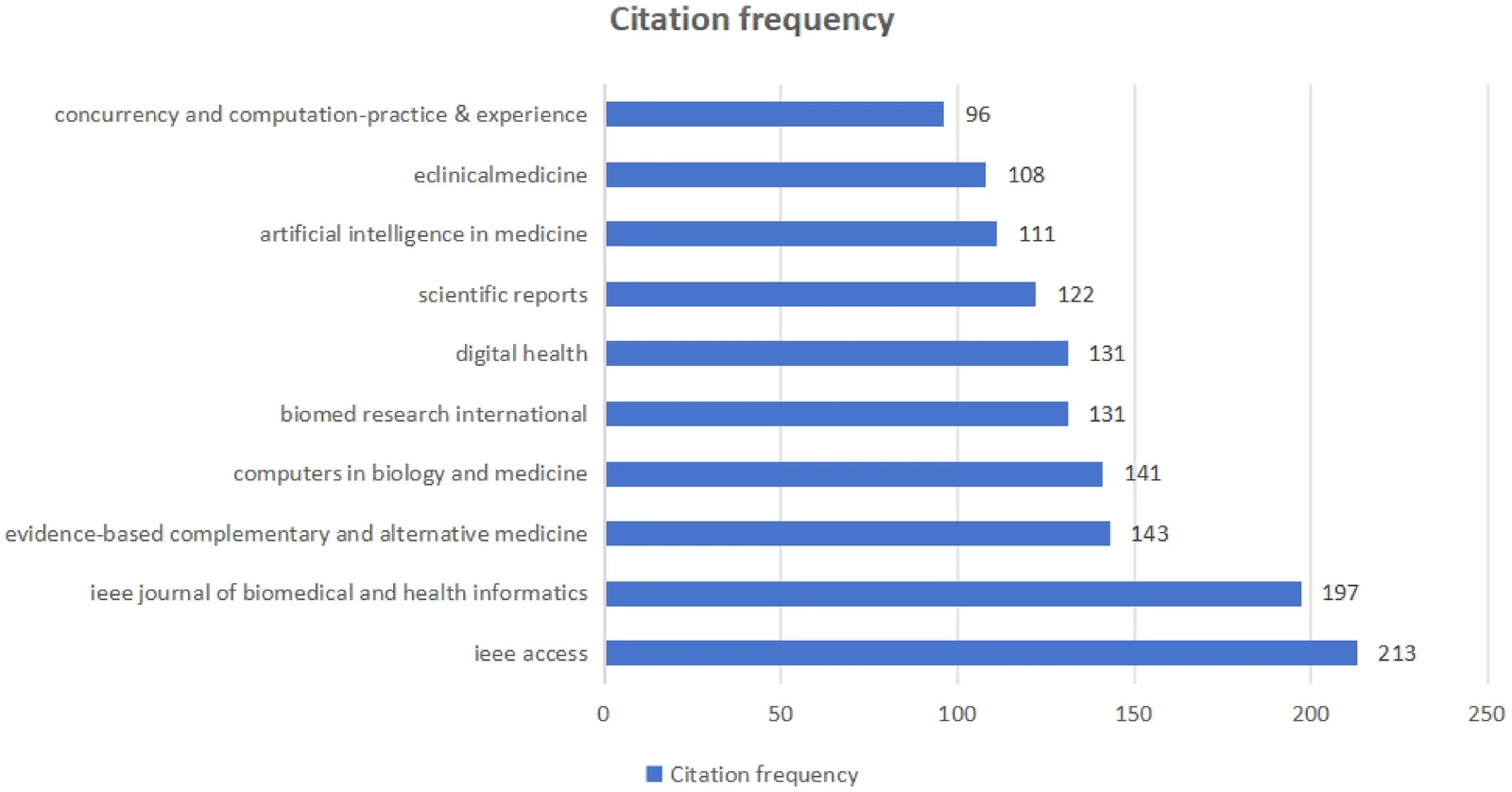
Top 10 most frequently cited journals in the WoSCC dataset. Bars represent the citation frequency of each journal based on the reference data of the included WoSCC publications.

### 3.7 Analysis of highly cited publications

Citation analysis was performed to identify the most highly cited publications among the included records. Citation counts reflect the citation visibility of individual publications within a database; however, they are influenced by publication age, journal visibility, research-network structure, and field-specific citation practices and should not be interpreted as direct measures of methodological quality. Because citation counts in WoSCC and CNKI are derived from different indexing and citation-tracking systems, they were interpreted within each database rather than directly compared across databases.

The top ten most cited publications among the included WoSCC records are presented in **Table 4**. The most cited article was Xu et al. (2020), which proposed a multi-task joint learning model for tongue-image segmentation and classification using a deep neural network, with 169 total citations. This was followed by Wang et al. (2020), who applied a deep convolutional neural network to tooth-marked tongue recognition (88 citations), and Zhang et al. (2017), who developed a support vector machine-based diagnostic method for diabetes using tongue images (68 citations). Other highly cited studies addressed tongue recognition, tongue constitution classification, tongue localization and segmentation, image-quality assessment, and disease-related applications such as non-alcoholic fatty liver disease and diabetes. When citation counts were expressed as citations per year, Xu et al. (2020) also had the highest citation rate (28.17 citations/year), followed by Wang et al. (2020) (14.67 citations/year) and Li et al. (2022) (11.50 citations/year) (**Table 4**).

**Table 4:** Top 10 most cited publications in the WoSCC dataset, ranked by total citations.

Rank	First author	Year	Title	Journal	Country region	Total citations	Citations per year
1	Xu Q	2020	Multi-task joint learning model for segmenting and classifying tongue images using a deep neural network	IEEE Journal of Biomedical and Health Informatics	China	169	28.17
2	Wang X	2020	Artificial intelligence in tongue diagnosis: using deep convolutional neural network for recognizing unhealthy tongue with tooth-mark	Computational and Structural Biotechnology Journal	China	88	14.67
3	Zhang JF	2017	Diagnostic method of diabetes based on support vector machine and tongue images	BioMed Research International	China	68	7.56
4	Li XQ	2018	Tooth-marked tongue recognition using multiple instance learning and CNN features	IEEE Transactions on Cybernetics	China	57	7.13
5	Ma JJ	2019	Complexity perception classification method for tongue constitution recognition	Artificial Intelligence in Medicine	China	51	7.29
6	Zhou C	2019	TongueNet: accurate localization and segmentation for tongue images using deep neural networks	IEEE Access	China	50	7.14
7	Jiang T	2021	Tongue image quality assessment based on a deep convolutional neural network	BMC Medical Informatics and Decision Making	China	50	10.00
8	Jiang T	2021	Application of computer tongue image analysis technology in the diagnosis of NAFLD	Computers in Biology and Medicine	China	48	9.60
9	Li J	2022	A multi-step approach for tongue image classification in patients with diabetes	Computers in Biology and Medicine	China	46	11.50
10	Zhou J	2019	TongueNet: a precise and fast tongue segmentation system using U-Net with a morphological processing layer	Applied Sciences	China	46	6.57

Articles were ranked by total citation count in WoSCC.Citations per year were calculated as total citations divided by the number of years from publication to 2026.

The top ten most cited publications among the included CNKI records are presented in **Table 5**. The most cited article was Liu et al. (2019), which investigated tongue-image extraction and recognition using deep learning and transfer learning, with 119 total citations. This was followed by Li et al. (2017), who reviewed the development and application of tongue diagnostic instruments in objective tongue diagnosis research (83 citations), and Li et al. (2022), who analysed tongue-image features using deep learning (74 citations). Other highly cited CNKI publications focused on objective tongue diagnosis based on digital image processing, artificial intelligence-assisted tongue diagnosis, tongue-image recognition for TCM syndrome classification, tongue-image segmentation, and TCM constitution identification. When adjusted for publication year, Liu et al. (2019) had the highest citation rate (17.00 citations/year), followed by Jiang et al. (2022) (15.75 citations/year) and Li et al. (2022) (12.33 citations/year) (**Table 5**).

**Table 5:** Top 10 most cited publications in the CNKI dataset, ranked by total citations.

Rank	First author	Year	Title	Journal	Country/region	Total citations	Citations per year
1	Liu M	2019	Research on extraction and recognition of TCM tongue images based on deep learning and transfer learning	Journal of Traditional Chinese Medicine	China	119	17.00
2	Li DX	2017	Development of tongue diagnostic instruments and their application in objective tongue diagnosis research	World Chinese Medicine	China	83	9.22
3	Li YT	2020	Tongue image feature analysis based on deep learning	Computer Science	China	74	12.33
4	Qi Z	2015	Progress in the clinical application of objective tongue diagnosis based on digital image processing technology	China Journal of Traditional Chinese Medicine and Pharmacy	China	66	6.00
5	Jiang ZQ	2022	Overview of objective research on TCM tongue diagnosis in the context of artificial intelligence	Computer Era	China	63	15.75
6	Kan HX	2016	A tongue image recognition method for TCM syndrome types of type 2 diabetes mellitus	Chinese Journal of Biomedical Engineering	China	63	6.30
7	Lu YX	2021	Research progress in TCM tongue image segmentation technology: methods, performance, and prospects	Acta Automatica Sinica	China	58	11.60
8	Pan SX	2020	Research on a TCM constitution identification model based on neural networks and support vector machines	World Science and Technology–Modernization of Traditional Chinese Medicine	China	57	9.50
9	Fan W	2019	Application of artificial intelligence in TCM tongue diagnosis	Guangming Journal of Chinese Medicine	China	55	7.86
10	Ma LX	2020	Research on a tongue image segmentation algorithm based on high-resolution features	Computer Engineering	China	52	8.67

Citation counts were obtained from CNKI and reflect citation visibility within the CNKI database. They should not be directly compared with citation counts from WoSCC because the two databases use different indexing and citation-tracking systems. Citations per year were calculated as total citations divided by the number of years from publication to 2026. Chinese article titles, author names, and journal names were translated into English for presentation; the original Chinese bibliographic information is provided in **Supplementary Table S3.**

### 3.8 Keyword analysis

#### 3.8.1 Keyword co-occurrence analysis

Keyword co-occurrence analysis was performed using CiteSpace 6.4.R1 to identify major research topics and their interrelationships. Betweenness centrality reflects the extent to which a keyword occupies a bridging position within the network, with higher values indicating a stronger potential role in connecting different parts of the network (Cheng et al., 2021). **Table 6** presents the 15 most frequent keywords in each dataset, and **Figure 7** shows the corresponding co-occurrence networks.

**Table 6:** Top 15 high-frequency keywords in the CNKI and WoSCC datasets.

Rank	CNKI keyword	Frequency	Centrality	WoSCC keyword	Frequency	Centrality
1	tongue diagnosis	56	0.61	traditional chinese medicine	91	0.10
2	deep learning	44	0.08	deep learning	73	0.04
3	TCM tongue diagnosis	37	0.32	tongue diagnosis	68	0.28
4	artificial intelligence	30	0.26	diagnosis	60	0.22
5	tongue image	21	0.38	tongue image	50	0.30
6	machine learning	18	0.24	machine learning	39	0.11
7	objectification	17	0.46	tongue segmentation	37	0.10
8	feature extraction	15	0.16	image classification	34	0.18
9	image segmentation	9	0.20	colour	28	0.14
10	feature fusion	8	0.10	convolutional neural network	27	0.14
11	image processing	7	0.40	diabetes mellitus	18	0.50
12	TCM diagnosis	7	0.24	images	16	0.18
13	tongue body segmentation	7	0.08	artificial intelligence	15	0.11
14	neural network	6	0.22	image segmentation	15	0.04
15	facial diagnosis	6	0.25	feature extraction	13	0.24

Centrality refers to betweenness centrality. CNKI, China National Knowledge Infrastructure; WoSCC, Web of Science Core Collection.

**Figure 7:**
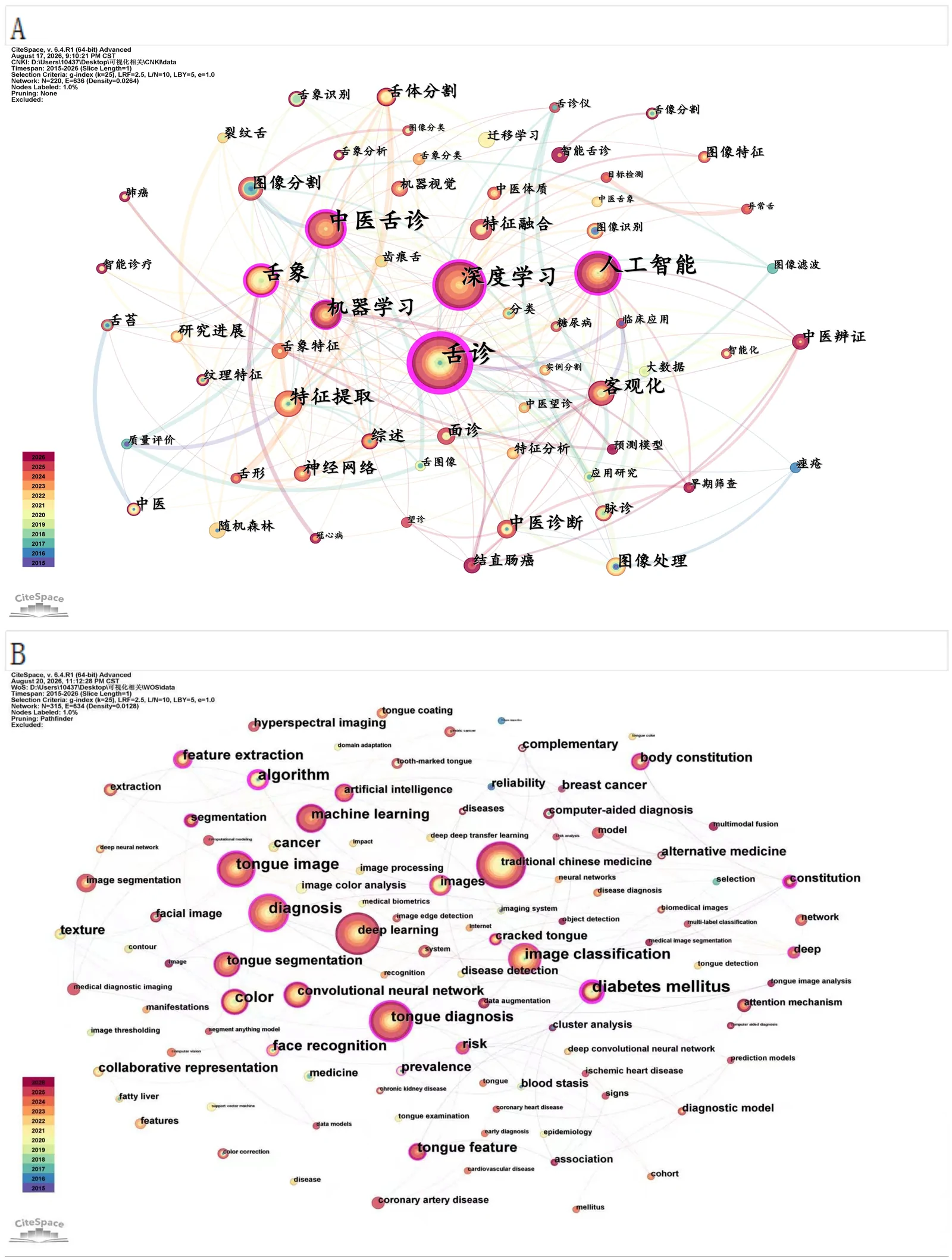
Keyword co-occurrence networks in the CNKI- and WoSCC-indexed literature. **(A)** CNKI-indexed literature; **(B)** WoSCC-indexed literature. Node size represents keyword frequency, links indicate keyword co-occurrence, and colours indicate the temporal distribution of keywords. Purple rings indicate nodes with betweenness centrality ≥0.10.

In the CNKI-indexed literature, the keyword network consisted of 220 nodes and 636 links, with a network density of 0.0264. “Tongue diagnosis” was the most frequent keyword (frequency = 56) and also showed the highest betweenness centrality (0.61). Other frequently occurring keywords included “deep learning” (44), “TCM tongue diagnosis” (37), “artificial intelligence” (30), “tongue image” (21), and “machine learning” (18). In addition to “tongue diagnosis,” relatively high betweenness centrality was observed for “objectification” (0.46), “tongue image” (0.38), “TCM tongue diagnosis” (0.32), and “artificial intelligence” (0.26). Technical keywords such as “feature extraction” (frequency = 15), “image segmentation” (9), and “feature fusion” (8) were also represented in the network. These results indicate that the CNKI-indexed literature is characterized by the co-occurrence of TCM tongue-diagnosis concepts and computational approaches related to image analysis and artificial intelligence.

In the WoSCC-indexed literature, the network contained 315 nodes and 634 links, with a density of 0.0128. “Traditional Chinese medicine” was the most frequently occurring keyword (frequency = 91), followed by “deep learning” (73), “tongue diagnosis” (68), “diagnosis” (60), and “tongue image” (50). Among the 15 most frequent keywords, “diabetes mellitus” showed the highest betweenness centrality (0.50), followed by “tongue image” (0.30) and “tongue diagnosis” (0.28). Methodological terms, including “machine learning” (frequency = 39), “tongue segmentation” (37), “image classification” (34), and “convolutional neural network” (27), were also prominent. The network therefore reflects the concurrent presence of TCM-related concepts, tongue-image analysis, and AI-based image-processing methods in the WoSCC-indexed literature.

Overall, both datasets showed recurring links between tongue-diagnosis concepts and AI-related image-analysis methods, although their keyword frequency and centrality distributions differed. Because the two datasets differ in database coverage, indexing practices, and corpus size, these differences are interpreted as database-specific bibliometric patterns rather than direct evidence of differences between Chinese- and English-language research traditions.

#### 3.8.2 Keyword cluster analysis

Keyword clustering analysis was conducted using CiteSpace 6.4.R1 (**Figure 8**). Clustering groups related keywords to characterize the thematic structure of a research field (Chen et al., 2023). A modularity Q value greater than 0.3 generally indicates meaningful separation between clusters, while a mean silhouette S value greater than 0.5 indicates acceptable within-cluster consistency and a value greater than 0.7 indicates high consistency (Chen et al., 2015). Detailed characteristics of the major keyword clusters, including the original cluster labels, standardized English translations, cluster size, silhouette value, mean year, and representative terms, are provided in **Supplementary Table S4**.

**Figure 8:**
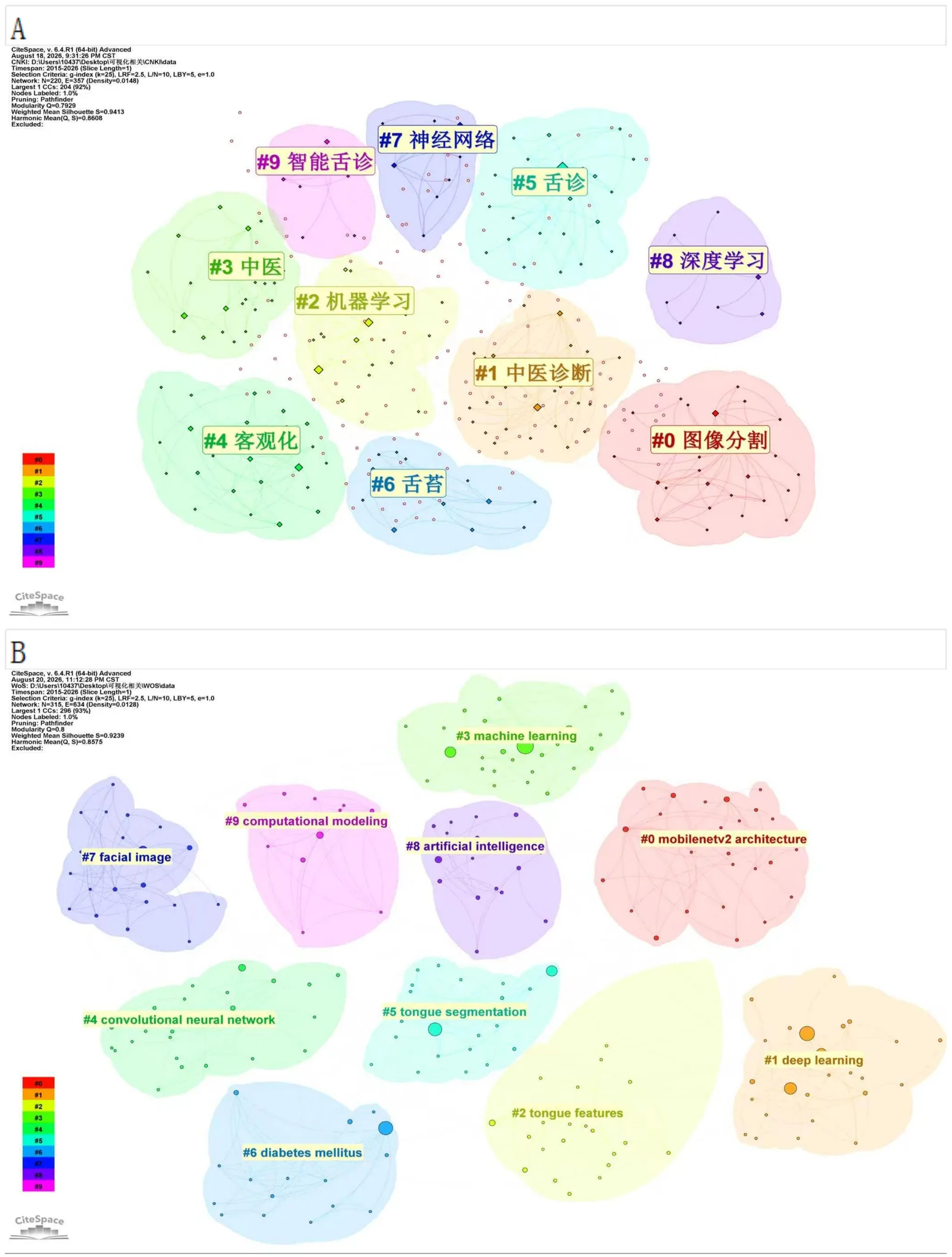
Keyword clustering networks in the CNKI- and WoSCC-indexed literature. **(A)** CNKI-indexed literature; **(B)** WoSCC-indexed literature. coloured regions represent keyword clusters; cluster numbers are assigned in descending order of cluster size, with #0 representing the largest cluster.

The CNKI keyword network comprised 220 nodes and 357 links, with a network density of 0.0148. The clustering solution yielded a modularity Q of 0.7929 and a weighted mean silhouette S of 0.9413, indicating well-defined and internally consistent clusters. The ten major clusters shown in **Figure 8A** were #0 image segmentation, #1 TCM diagnosis, #2 machine learning, #3 traditional Chinese medicine, #4 objectification, #5 tongue diagnosis, #6 tongue coating, #7 neural network, #8 deep learning, and #9 intelligent tongue diagnosis. These clusters mainly represented three interconnected thematic domains: TCM diagnostic concepts and objectification, tongue and tongue-coating characterization, and computational methods for tongue-image analysis.

The WoSCC keyword network comprised 315 nodes and 634 links, with a network density of 0.0128. The clustering solution showed a modularity Q of 0.8000 and a weighted mean silhouette S of 0.9239, likewise indicating clear separation and high within-cluster consistency. The ten major clusters displayed in **Figure 8B** were #0 MobileNetV2 architecture, #1 deep learning, #2 tongue features, #3 machine learning, #4 convolutional neural network, #5 tongue segmentation, #6 diabetes mellitus, #7 facial image, #8 artificial intelligence, and #9 computational modelling. These clusters primarily represented AI architectures and computational methods, tongue-feature extraction and segmentation, and disease-related applications.

Overall, both datasets contained thematic structures related to AI-assisted tongue-image analysis, but their distributions differed. CNKI-indexed literature showed greater prominence of TCM diagnostic concepts and objectification alongside computational methods, whereas WoSCC-indexed literature showed more differentiated clusters related to AI architectures, computational modelling, tongue-feature analysis, and disease-oriented applications. Given differences in database coverage, indexing practices, and corpus composition, these findings are interpreted as database-specific thematic patterns. Detailed characteristics of all keyword clusters, including cluster size, silhouette value, mean year, and representative terms, are provided in **Supplementary Table S4**.

#### 3.8.3 Keyword clustering timeline

The temporal evolution of the major keyword clusters was further visualized using the timeline function in CiteSpace 6.4.R1 (**Figure 9**). The timeline maps display the occurrence and persistence of keywords within each cluster from 2015 to 2026, allowing changes in research emphasis over time to be examined.

**Figure 9:**
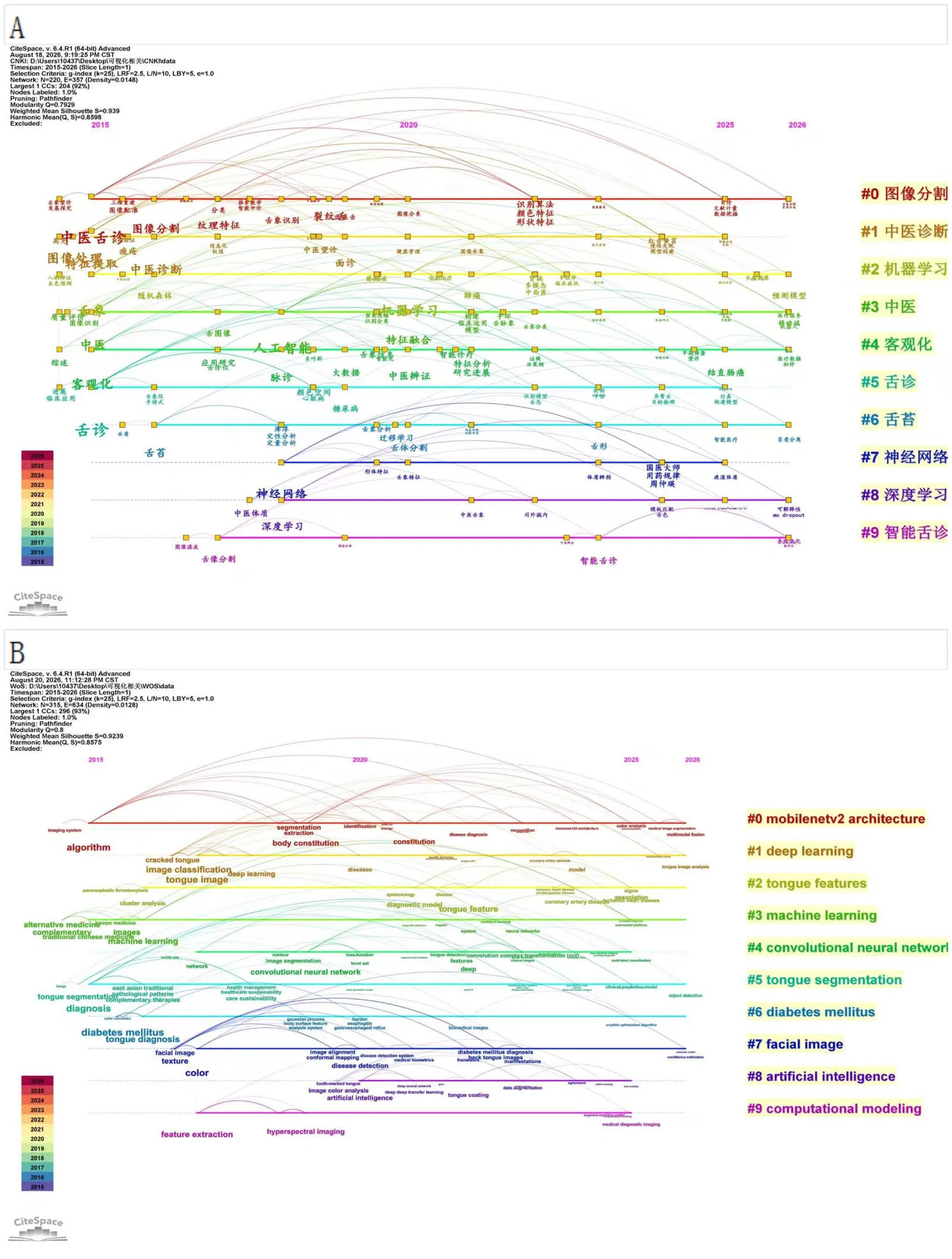
Keyword clustering timeline maps in the CNKI- and WoSCC-indexed literature. **(A)** CNKI-indexed literature; **(B)** WoSCC-indexed literature. Each horizontal line represents a keyword cluster; nodes represent keywords and their positions indicate the temporal distribution of keyword occurrences.

In the CNKI-indexed literature, earlier research was characterized by terms related to TCM tongue diagnosis, tongue-image objectification, image feature extraction, neural networks, and image segmentation. During the middle period, machine learning, artificial intelligence, feature fusion, tongue-coating analysis, transfer learning, and disease-related applications became increasingly represented. In more recent years, terms such as intelligent tongue diagnosis, prediction model, multimodal fusion, and medical data mining appeared across active clusters, indicating a progressive expansion toward more integrated AI-assisted analytical approaches.

In the WoSCC-indexed literature, earlier research included terms such as algorithm, tongue image, image classification, tongue segmentation, feature extraction, colour, and conventional machine-learning methods. Over time, deep learning, convolutional neural networks, diagnostic modelling, tongue-feature analysis, and disease-related applications became increasingly prominent. In the more recent period, terms related to artificial intelligence, data augmentation, multimodal fusion, and medical image analysis were represented across several active clusters, while segmentation and classification remained persistent methodological themes.

Taken together, the timeline maps show a temporal progression in both datasets from conventional tongue-image processing and manually oriented feature characterization toward increasingly diverse machine-learning, deep-learning, modelling, and multimodal approaches. The persistence of segmentation and feature-analysis themes across multiple periods also suggests that these foundational tasks continued to underpin more recent AI-assisted tongue diagnosis research.

#### 3.8.4 Keyword burst analysis

The original Chinese burst terms and their standardised English translations are provided in **Supplementary Table S5**. Keyword burst detection was performed using CiteSpace 6.4.R1 to identify keywords that experienced a marked increase in research attention during specific periods (**Figure 10**). A keyword burst reflects a temporary increase in the occurrence of a term within a defined time interval, with burst strength indicating the intensity of this increase.

**Figure 10:**
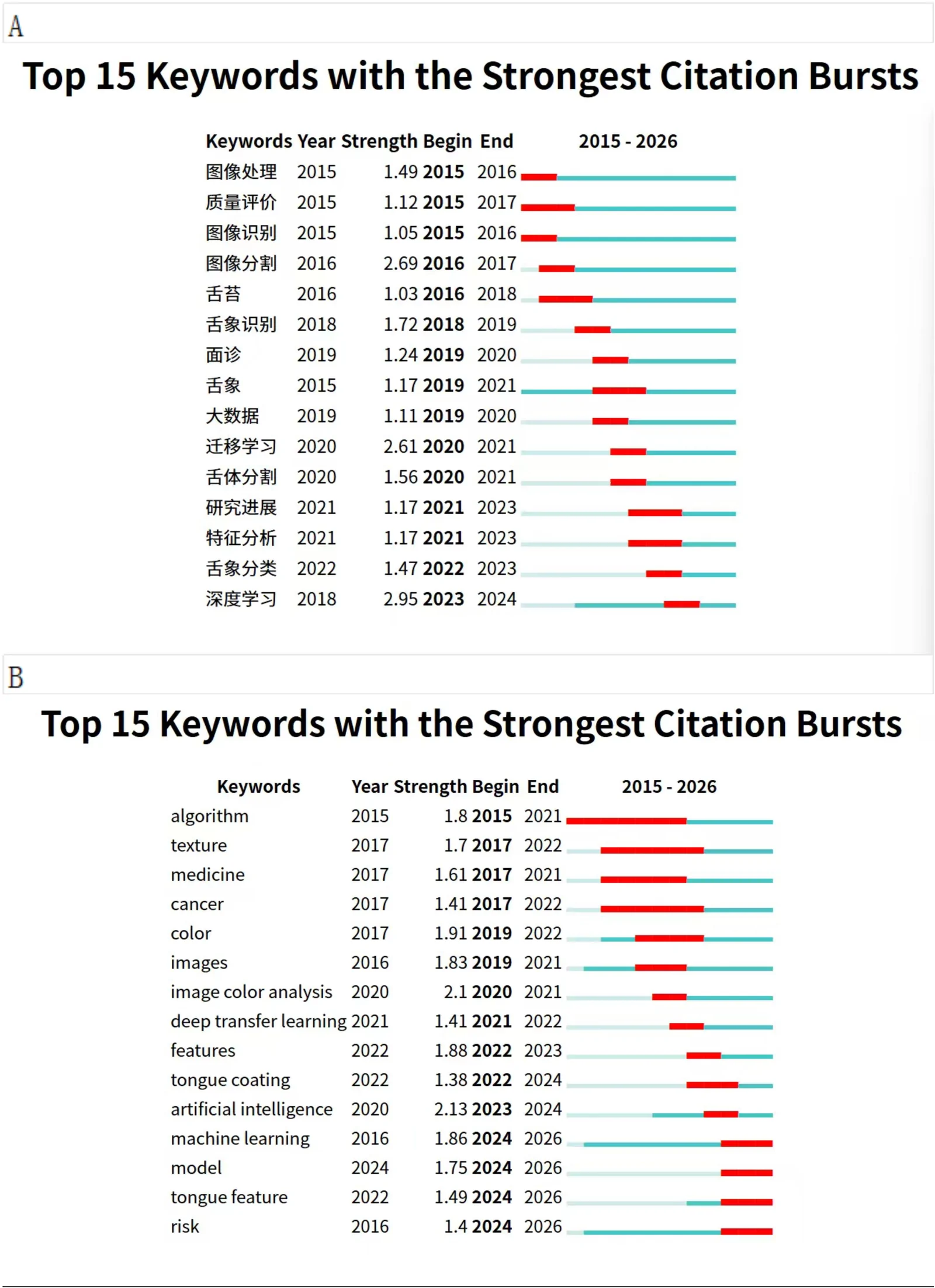
Top 15 keywords with the strongest citation bursts in the CNKI- and WoSCC-indexed literature. **(A)** CNKI-indexed literature; **(B)** WoSCC-indexed literature. Strength represents burst intensity, while Begin and End indicate the start and end years of the burst period. Red segments indicate periods of active keyword bursts.

In the CNKI-indexed literature, 15 keywords with the strongest citation bursts were identified (**Figure 10A**). The strongest burst was observed for “deep learning” (strength = 2.95; 2023–2024), followed by “image segmentation” (2.69; 2016–2017) and “transfer learning” (2.61; 2020–2021). Early bursts were mainly associated with image-related techniques, including “image processing” (1.49; 2015–2016), “image recognition” (1.05; 2015–2016), and “image segmentation” (2.69; 2016–2017), as well as “quality evaluation” (1.12; 2015–2017). Subsequent burst terms included “tongue coating” (1.03; 2016–2018), “tongue-image recognition” (1.72; 2018–2019), “tongue image” (1.17; 2019–2021), “big data” (1.11; 2019–2020), “transfer learning” (2.61; 2020–2021), and “tongue segmentation” (1.56; 2020–2021). More recent bursts included “research progress” and “feature analysis” (both strength = 1.17; 2021–2023), “tongue-image classification” (1.47; 2022–2023), “deep learning” (2.95; 2023–2024) and “facial diagnosis” (1.24; 2019–2020). No burst keyword in the CNKI dataset remained active through 2026.

In the WoSCC-indexed literature, 15 keywords with the strongest citation bursts were also identified (**Figure 10B**). “Artificial intelligence” showed the strongest burst (strength = 2.13; 2023–2024), followed by “image colour analysis” (2.10; 2020–2021), “colour” (1.91; 2019–2022), “features” (1.88; 2022–2023), and “machine learning” (1.86; 2024–2026). “Algorithm” exhibited the longest burst duration, extending from 2015 to 2021 (strength = 1.80). Earlier and intermediate burst terms included “texture” (1.70; 2017–2022), “medicine” (1.61; 2017–2021), “cancer” (1.41; 2017–2022), “images” (1.83; 2019–2021), and “deep transfer learning” (1.41; 2021–2022). More recent bursts involved “tongue coating” (1.38; 2022–2024) and “artificial intelligence” (2.13; 2023–2024). Notably, “machine learning” (1.86), “model” (1.75), “tongue feature” (1.49), and “risk” (1.40) all showed bursts extending from 2024 through 2026, indicating that these terms remained active at the end of the study period.

Overall, the burst patterns show a temporal shift in prominent keywords from image processing, recognition, colour, and texture-related topics toward machine learning, deep learning, transfer learning, modelling, and tongue-feature analysis. The persistence of several WoSCC burst terms through 2026 indicates continuing bibliometric attention to these topics, although keyword bursts reflect changes in research activity rather than evidence of technological maturity or clinical effectiveness.

## 4 Discussion

### 4.1 Principal findings and comparison with previous bibliometric studies

This study provides an updated comparative overview of AI-assisted tongue diagnosis research from 2015 to 2026 based on 482 eligible publications, including 187 CNKI-indexed Chinese-language records and 295 WoSCC-indexed English-language records. Publication output increased in both datasets, particularly after approximately 2020, with higher recent annual output in WoSCC. More importantly, the two datasets showed shared technological development but different thematic emphases. CNKI-indexed studies gave greater prominence to tongue-diagnosis objectification, image segmentation, TCM diagnostic concepts, and intelligent tongue diagnosis, whereas WoSCC-indexed studies showed greater differentiation of AI architectures, tongue-feature analysis, computational modelling, and disease-related applications.

These findings are broadly consistent with previous bibliometric studies but extend them in several respects. Tian et al. identified rapid growth of AI research across the four TCM diagnostic methods and highlighted deep-neural-network-based tongue-image classification and diagnosis as important hotspots (Tian et al., 2023). Zhang et al., using CNKI, Wanfang, VIP, and WoSCC, similarly identified neural networks, machine learning, image processing, feature recognition, and tongue diagnosis as prominent themes in AI-assisted TCM diagnosis (Zhang et al., 2023). However, that study addressed AI in TCM diagnosis broadly rather than tongue diagnosis specifically. More recently, Liu et al. analysed 198 WoSCC publications on AI-assisted tongue diagnosis and identified four thematic domains involving AI architectures, image processing and segmentation, TCM-specific applications, and disease-specific applications (Liu et al., 2026). The present study therefore confirms these broader trends while extending the evidence base to August 2026 and, importantly, comparing CNKI-indexed and WoSCC-indexed literature as separate bibliometric corpora.

The observed differences should not be interpreted simply as cultural differences between “Chinese” and “international” research. Database coverage, indexing policies, journal selection, metadata conventions, and language-specific publication practices are closely linked to the two datasets. Accordingly, our findings are better understood as database-specific but complementary patterns that together provide a broader view of the development of AI-assisted tongue diagnosis.

### 4.2 Collaboration structure and research capacity

The collaboration analyses suggest that AI-assisted tongue diagnosis has developed around several relatively stable research groups and institutions, although the degree of connectivity differed between the two datasets. The WoSCC author network was somewhat denser than the CNKI network, and the difference was more apparent at the institutional level, where network density was 0.0222 for WoSCC and 0.0101 for CNKI. Shanghai University of Traditional Chinese Medicine was prominent in both datasets, and China occupied the leading position in the WoSCC country network in both publication frequency and betweenness centrality.

These findings are consistent with previous studies. Tian et al. identified China as the leading contributor and Shanghai University of Traditional Chinese Medicine as a major institution in AI research involving the four TCM diagnostic methods (Tian et al., 2023). Liu et al. likewise reported that China accounted for the majority of affiliations in their WoSCC corpus, with Shanghai University of Traditional Chinese Medicine and Jiatuo Xu among the most productive contributors (Liu et al., 2026). This concentration of research capacity is also reflected in the highly cited literature, as several influential studies on tongue-image quality control, NAFLD assessment, and diabetes-related tongue classification originated from research teams affiliated with Shanghai University of Traditional Chinese Medicine (Jiang et al., 2021a, Jiang et al., 2021b; Li et al., 2022). The recurrence of these centres across productivity, collaboration, and citation analyses suggests sustained research capacity rather than isolated short-term activity.

However, productive core groups do not necessarily imply broad cross-institutional or international integration. This is relevant because limitations in dataset construction and diversity, variability in image acquisition and quality, and restricted generalizability remain important challenges in tongue-image AI (Jiang et al., 2021a; Liu et al., 2023; Li et al., 2025). Future collaboration should therefore move beyond co-authorship toward shared datasets, harmonized acquisition and annotation protocols, independent external validation, and multicentre studies. Such collaboration may ultimately be more important for reproducibility and clinical translation than continued increases in publication volume alone.

### 4.3 Evolution of research themes

Keyword clustering, timeline mapping, and burst detection together suggest changes in the thematic focus of AI-assisted tongue diagnosis over time. CNKI clusters emphasized image segmentation, TCM diagnosis, objectification, tongue coating, machine learning, deep learning, and intelligent tongue diagnosis. In contrast, WoSCC showed more differentiated method-oriented clusters, including MobileNetV2, convolutional neural networks, tongue features, tongue segmentation, computational modelling, and a distinct diabetes mellitus cluster.

Temporally, research in both datasets progressed from image processing, colour and texture analysis, feature extraction, and segmentation toward machine learning, deep learning, transfer learning, feature fusion, diagnostic modelling, and multimodal approaches. In CNKI, deep learning, image segmentation, and transfer learning showed prominent bursts, whereas machine learning, model, tongue feature, and risk remained active topics in WoSCC through 2026. These differences should not be interpreted as evidence that one literature is technologically more advanced; rather, they may reflect differences in terminology, indexing, timing, and dissemination between the two publication environments.

The prominence of segmentation in the temporal and clustering analyses is also reflected in the highly cited literature. Influential studies developed automated tongue-localization and segmentation frameworks using multi-task learning, multi-scale feature extraction, U-Net architectures, and morphological refinement (Zhou et al., 2019; Zhou et al., 2019). Together with the multi-task segmentation-classification framework developed by Xu et al. (2020), these studies indicate that reliable tongue-region extraction represented an important technical foundation for downstream feature analysis and diagnostic modelling.

This transition is consistent with the review by Liu et al., which described a progression from conventional feature-based approaches toward deep-learning methods for calibration, detection, segmentation, disease classification, and syndrome differentiation (Liu et al., 2023). More recent work has increasingly explored attention mechanisms, multiscale representation, multimodal fusion, self-supervised learning, and Transformer architectures (Li et al., 2022; Liu et al., 2023, 2026). Beyond the bibliometric findings of the present study, recent methodological developments in related visual and medical-imaging fields may provide additional technical directions for future tongue-image analysis. These include multi-level attention fusion, hybrid CNN–Transformer architectures, and attention-based boundary-refinement strategies, which may offer testable approaches for improving tongue-image segmentation (Ali et al., 2026; Ji et al., 2026; Khan and Khan, 2026). However, because these approaches were developed in other visual or medical-imaging domains, their applicability to tongue images requires direct validation rather than assumed transferability. More broadly, visual-intelligence research highlights the importance of robustness, computational efficiency, and deployment-relevant evaluation beyond benchmark accuracy (Zeb et al., 2026). Recent advances in real-time optimization further illustrate the potential value of computationally efficient algorithms for resource-constrained applications (Lin, 2026).

Importantly, bibliometric emergence should not be equated with technological maturity. Keyword bursts indicate increasing research attention rather than proven diagnostic superiority or clinical readiness.

### 4.4 From foundational image processing to disease-oriented modelling

The highly cited literature illustrates three interconnected research directions: foundational tongue segmentation and feature extraction, image standardization and quality control, and increasingly disease-oriented modelling. Xu et al. (2020), whose publication was the most cited in the included WoSCC corpus, integrated tongue segmentation and classification through multi-task deep learning. Other highly cited studies developed TongueNet architectures combining localization and segmentation (Zhou C. et al., 2019) or U-Net with morphological refinement (Zhou J. et al., 2019). These studies collectively reinforce the importance of accurate tongue-region extraction before quantitative phenotype characterization or diagnostic prediction.

A similar methodological emphasis was observed in the highly cited CNKI literature. Liu et al. (2019), whose publication was the most cited in the included CNKI corpus, applied deep learning and transfer learning to tongue-image extraction and recognition, while Lu et al. systematically examined advances in tongue-image segmentation methods. These findings suggest that segmentation and feature extraction have also formed an important methodological foundation of the Chinese-language literature (Lu et al., 2021).

A second development involves standardization of the input data itself. Jiang et al. developed a deep-CNN-based tongue-image quality assessment framework to automatically distinguish qualified from poor-quality images, emphasizing that standardized datasets require not only consistent acquisition protocols but also systematic image-quality control (Jiang et al., 2021a). The CNKI literature also highlighted the importance of standardized acquisition. Li et al. (2017) reviewed the development and application of tongue diagnostic instruments as a basis for improving the objectification and reproducibility of tongue diagnosis. This represents an important intermediate step between image acquisition and downstream modelling: even sophisticated algorithms may be compromised when exposure, illumination, focus, tongue posture, or other acquisition-related factors are inadequately controlled.

Disease-specific applications were also represented among the highly cited publications. Jiang et al. combined quantitative tongue-image parameters with anthropometric and serological variables in modelling NAFLD; their best fusion model incorporated tongue features together with waist circumference, BMI, and biochemical indicators, illustrating a shift from tongue-image-only analysis toward integration with conventional clinical information (Jiang et al., 2021b). Li et al. adopted a different strategy for diabetes, combining VQ-VAE representation learning, K-means clustering, Vision Transformer classification, and Grad-CAM visualization to achieve data-driven fine classification of diabetic tongue images (Li et al., 2022). Disease-oriented modelling was also evident among the highly cited CNKI publications. Kan et al. (2016) used tongue-image features and a random forest classifier to identify TCM syndrome patterns in patients with type 2 diabetes, reflecting an earlier effort to extend objective tongue-image analysis from feature recognition toward clinically defined disease and syndrome classification. Together, these studies suggest that influential research has moved beyond isolated feature recognition toward standardized data pipelines, phenotype characterization, integrated modelling, and disease-oriented applications.

Nevertheless, bibliometric visibility and promising model performance do not establish clinical effectiveness. The NAFLD investigators themselves emphasized that tongue images should not serve as the primary basis for clinical diagnosis and that further clinical verification was needed (Jiang et al., 2021b). Similarly, the diabetes classification study acknowledged its relatively limited dataset and the need to improve visualization and further validate the inferred patterns (Li et al., 2022). Liu et al. also reported substantial heterogeneity in their separate diagnostic-test-accuracy meta-analysis of AI-assisted tongue diagnosis (Liu et al., 2026). Thus, publication growth, citation impact, and high reported performance should be interpreted as indicators of research activity and methodological development rather than proof that AI-assisted tongue diagnosis is ready for routine practice.

### 4.5 Practical implications

The bibliometric patterns identified in this study suggest several priorities for the future development of AI-assisted tongue diagnosis. Greater emphasis should be placed on standardized tongue-image acquisition and annotation, reproducible phenotype definitions, robust image-quality control, multimodal modelling, external validation, and clinically interpretable outputs. Closer integration of TCM-oriented phenotype characterization with quantitative computational modelling may help improve the reproducibility, generalizability, and clinical interpretability of AI-assisted tongue diagnosis systems.

### 4.6 Strengths, limitations, and future directions

Several strengths of this study should be noted. First, CNKI- and WoSCC-indexed literature were analysed as separate but complementary bibliographic corpora, allowing database-specific patterns to be examined without directly conflating differences in indexing and citation systems. Second, both searches were updated to August 2026, and the broader WoSCC search strategy was quantitatively evaluated against a more TCM-specific strategy. Third, predefined eligibility criteria, independent screening, explicit data-cleaning procedures, and complementary bibliometric tools were applied to enhance the transparency and reproducibility of the analysis.

Several limitations should be considered. First, only CNKI and WoSCC were included, so studies indexed exclusively in PubMed, Scopus, Wanfang, VIP, or other databases may have been missed. Second, database and language effects cannot be separated because the CNKI corpus consisted of Chinese-language journal articles whereas the WoSCC corpus consisted of English-language articles and reviews. Differences in coverage, indexing rules, metadata structures, citation practices, and publication conventions may therefore contribute to the observed patterns.

Third, conference proceedings were excluded to improve consistency in publication type and metadata. Because conferences are important dissemination routes in computer science and AI, some recent methodological developments may consequently be underrepresented. Fourth, bibliometric indicators describe publication activity, citation visibility, collaboration, and research attention but cannot directly assess methodological quality, risk of bias, diagnostic accuracy, or clinical effectiveness. Citation counts are also affected by publication age and journal visibility; citations per year reduce but do not eliminate this bias. Fifth, keyword-based retrieval may miss studies using alternative or emerging terminology, while keyword standardization and clustering involve analytical decisions that may influence network structure. Finally, 2026 represents only publications available up to the August search dates, and keyword bursts or network associations should not be interpreted as causal evidence or proof of technological maturity. Given the rapid evolution of AI technologies, the identified hotspots and emerging themes may change after the final search dates.

These limitations also define several priorities for future research. First, standardized multicentre tongue-image datasets are needed, with harmonized acquisition conditions, colour calibration, phenotype definitions, and annotation protocols. Importantly, standardization should include automated image-quality control because poor exposure, blurring, inappropriate illumination, contamination, or tongue-posture abnormalities may compromise subsequent feature extraction and modelling (Jiang et al., 2021a). Shared benchmark and multicentre datasets, together with standardized image acquisition, would facilitate more consistent algorithm comparison and stronger assessment of robustness across acquisition settings (Jiang et al., 2021a; Liu et al., 2023; Li et al., 2025).

Second, model-development studies should emphasize independent external validation and, where appropriate, prospective clinical evaluation. Evaluation should extend beyond accuracy or AUC to calibration, clinically meaningful sensitivity and specificity, subgroup performance, failure analysis, workflow impact, and comparison with existing diagnostic approaches. For segmentation and preprocessing components, boundary accuracy, robustness to variations in tongue appearance and acquisition conditions, and computational efficiency should also be considered (Zhou J. et al., 2019; Zhou C. et al., 2019). TRIPOD+AI and DECIDE-AI provide complementary reporting guidance for prediction-model studies and early-stage live clinical evaluation of AI systems, respectively (Vasey et al., 2022; Collins et al., 2024).

Third, the emerging themes identified here suggest increasing interest in multimodal and integrated modelling (Jiang et al., 2021b; Li et al., 2022; Liu et al., 2023, 2026). Future TCM-oriented systems could combine quantitative tongue phenotypes with other components of the four diagnostic methods and conventional clinical information. Such models should remain clinically interpretable, with outputs linked to observable features and clearly defined clinical constructs. Human-centred explainable AI research likewise emphasizes that explanations should be designed around end-user needs and clinical context (Chen et al., 2022). International and cross-institutional collaboration will be important for determining whether these systems remain reliable across populations, imaging devices, and healthcare settings.

## 5 Conclusion

This bibliometric study provides an updated comparative overview of AI-assisted tongue diagnosis research from 2015 to August 2026 based on 482 publications from CNKI and WoSCC. The two bibliographic corpora showed complementary but database-specific research emphases. CNKI-indexed literature showed prominent links to tongue-image objectification, TCM diagnostic concepts, and intelligent tongue diagnosis, whereas WoSCC-indexed literature showed more differentiated themes involving AI architectures, computational modelling, tongue-feature analysis, and disease-oriented applications. Across both datasets, the bibliometric patterns and highly cited publications suggest a shift in research focus from conventional image processing, tongue-image acquisition, and feature extraction toward machine learning, deep learning, and increasingly integrated and disease-oriented AI applications. However, publication activity, citation visibility, and methodological sophistication should not be interpreted as evidence of clinical readiness or, more fundamentally, of the scientific and medical validity of AI-based tongue analysis. As a bibliometric review, this study characterizes research activity and trends but does not establish whether the underlying techniques are biologically plausible, diagnostically accurate, reproducible, or clinically useful. Future research may benefit from prioritizing standardised tongue-image acquisition and annotation, shared benchmark datasets, multicentre and independent external validation, explainable and clinically interpretable AI, stronger international and cross-institutional collaboration, and reproducible reporting standards. By examining CNKI- and WoSCC-indexed literature as separate but complementary corpora, this study provides a broader perspective on the development of AI-assisted tongue diagnosis while preserving database-specific differences and identifies priorities for future methodological integration and clinical validation.
