# Supplementary material for: Applications of artificial intelligence in traditional Chinese medicine tongue diagnosis: a bibliometric review of research trends and hotspots, 2015–2026
Source: Front Physiol. 2026 Oct 5;17:1895902. doi: 10.3389/fphys.2026.1895902 (PMC13678510; doi:10.3389/fphys.2026.1895902)
Supplement: Supplementary file 1 [file Table1.docx]

**Supplementary Table S1. Database-specific search strategies and retrieval parameters**

#### Panel A. CNKI search strategy

| **Search set** | **Search field** | **Search terms** |
| --- | --- | --- |
| #1 | **Subject (SU)** | 人工智能 + 机器智能 + 机器学习 + 深度学习 + 迁移学习 +人工神经网络 + 神经网络 + 卷积神经网络 +计算机视觉 + 机器视觉 + 图像识别 + 图像分类 +图像分割 + 图像处理 + 图像分析 + 模式识别 +特征提取 + 特征选择 + 支持向量机 + 随机森林 +决策树 + 贝叶斯网络 + 贝叶斯分类 + K近邻 +专家系统 + 模糊逻辑 + 数据挖掘 + 生成对抗网络 + Transformer + 视觉Transformer + 智能诊断 + 智能识别 |
| #2 | **Subject (SU)** | 舌象 + 舌像 + 舌诊 + 舌图像 + 舌象分析 + 舌图像分析 + 舌象识别 + 舌象分类 + 舌象分割 + 舌象特征 + 舌色 + 舌苔 + 舌质 + 舌体 + 舌形 + 舌形态 + 舌纹理 + 舌诊客观化 + 舌象客观化 |
| #3 | **Subject (SU)** | 中医 + 中医学 + 中医诊断 + 中医诊断学 + 辨证 + 辨证论治 + 证候 + 证型 +体质 + 中医体质 + 四诊 + 望诊 |
| #4 | — | #1 AND #2 AND #3 |

| **Item** | **Details** |
| --- | --- |
| Database | China National Knowledge Infrastructure (CNKI) |
| Time span | 1 January 2015 to 14 August 2026 |
| Document type | Journal article |
| Final search date | 14 August 2026 |
| Records retrieved | 297 |

**Panel B. WoSCC search strategy**

| **Search set** | **Search field** | **Search terms** |
| --- | --- | --- |
| #1 | Topic (TS) | (artificial intelligen* OR machine intelligen* OR machine learn* OR deep learn* OR transfer learn* OR artificial neural network* OR neural network* OR deep neural network* OR convolutional neural network* OR CNN OR computer vision OR machine vision OR image recognit* OR image classif* OR image segment* OR image process* OR image analys* OR pattern recognit* OR feature extract* OR feature select* OR support vector machine* OR SVM OR random forest* OR decision tree* OR Bayesian network* OR Bayes network* OR Bayesian classif* OR k nearest neighbor* OR KNN OR expert system* OR fuzzy logic OR data mining OR generative adversarial network* OR vision transformer* OR transformer*) |
| #2 | Topic (TS) | (tongue diagnos* OR tongue imag* OR tongue analys* OR tongue feature* OR tongue recognit* OR tongue segment* OR tongue classif* OR tongue color* OR tongue colour* OR tongue coating* OR tongue fur* OR tongue body OR tongue texture* OR tongue shape* OR tongue appearance* OR tongue inspection OR tongue assessment OR tongue morpholog* OR tongue characteristic* OR tongue manifestation*) |
| #3 | — | #1 AND #2 |

| **Item** | **Details** |
| --- | --- |
| Database | Web of Science Core Collection (WoSCC) |
| Time span | 1 January 2015 to 15 August 2026 |
| Language | English |
| Document type | Article or Review article |
| Final search date | 15 August 2026 |
| Records retrieved | 3716 |

| **Search set** | **Search field** | **Search terms** |
| --- | --- | --- |
| #1 | Topic (TS) | (artificial intelligen* OR machine intelligen* OR machine learn* OR deep learn* OR transfer learn* OR artificial neural network* OR neural network* OR deep neural network* OR convolutional neural network* OR CNN OR computer vision OR machine vision OR image recognit* OR image classif* OR image segment* OR image process* OR image analys* OR pattern recognit* OR feature extract* OR feature select* OR support vector machine* OR SVM OR random forest* OR decision tree* OR Bayesian network* OR Bayes network* OR Bayesian classif* OR k nearest neighbor* OR KNN OR expert system* OR fuzzy logic OR data mining OR generative adversarial network* OR vision transformer* OR transformer*) |
| #2 | Topic (TS) | (tongue diagnos* OR tongue imag* OR tongue analys* OR tongue feature* OR tongue recognit* OR tongue segment* OR tongue classif* OR tongue color* OR tongue colour* OR tongue coating* OR tongue fur* OR tongue body OR tongue texture* OR tongue shape* OR tongue appearance* OR tongue inspection OR tongue assessment OR tongue morpholog* OR tongue characteristic* OR tongue manifestation*) |
| #3 | Topic (TS) | traditional Chinese medicine OR TCM OR Chinese medicine OR Chinese medical diagnos* OR traditional Chinese medical diagnos* OR TCM diagnos* OR syndrome differentiation OR pattern differentiation OR pattern identification OR syndrome diagnos* OR pattern diagnos* OR syndrome classification OR TCM syndrome* OR Chinese medicine syndrome* OR body constitution OR tongue constitution* OR TCM constitution* OR Chinese medicine constitution* OR four diagnoses OR four examinations OR inspection diagnosis |
| #4 | — | #1 AND #2 AND #3 |

| **Item** | **Details** |
| --- | --- |
| Database | Web of Science Core Collection (WoSCC) |
| Time span | 1 January 2015 to 15 August 2026 |
| Language | English |
| Document type | Article or Review article |
| Final search date | 15 August 2026 |
| Records retrieved | 703 |

**Note:** SU denotes the Subject field in CNKI, and TS denotes the Topic field in WoSCC. The number of records retrieved refers to records obtained after applying the prespecified database-level limits and before title/abstract screening. CNKI, China National Knowledge Infrastructure; WoSCC, Web of Science Core Collection.

**Supplementary Table S2. Characteristics of the included bibliometric corpu**

| **Characteristic** | **Category** | **CNKI, n** | **CNKI, %** | **WoSCC, n** | **WoSCC, %** | **Total, n** | **Total, %** |
| --- | --- | --- | --- | --- | --- | --- | --- |
| Database source | CNKI | 187 | 100.0% | 0 | 0.0% | 187 | 38.8% |
|  | WoSCC | 0 | 0.0% | 295 | 100.0% | 295 | 61.2% |
| Language | Chinese | 187 | 100.0% | 0 | 0.0% | 187 | 38.8% |
|  | English | 0 | 0.0% | 295 | 100.0% | 295 | 61.2% |
| Document type | Journal article / Article | 187 | 100.0% | 291 | 98.6% | 478 | 99.2% |
|  | Review article | 0 | 0.0% | 4 | 1.4% | 4 | 0.8% |
| Publication year | 2015 | 8 | 4.3% | 4 | 1.4% | 12 | 2.5% |
|  | 2016 | 8 | 4.3% | 7 | 2.4% | 15 | 3.1% |
|  | 2017 | 8 | 4.3% | 12 | 4.1% | 20 | 4.1% |
|  | 2018 | 5 | 2.7% | 3 | 1.0% | 8 | 1.7% |
|  | 2019 | 11 | 5.9% | 15 | 5.1% | 26 | 5.4% |
|  | 2020 | 13 | 7.0% | 17 | 5.8% | 30 | 6.2% |
|  | 2021 | 22 | 11.8% | 28 | 9.5% | 50 | 10.4% |
|  | 2022 | 14 | 7.5% | 38 | 12.9% | 52 | 10.8% |
|  | 2023 | 21 | 11.2% | 29 | 9.8% | 50 | 10.4% |
|  | 2024 | 24 | 12.8% | 41 | 13.9% | 65 | 13.5% |
|  | 2025 | 31 | 16.6% | 69 | 23.4% | 100 | 20.7% |
|  | 2026 | 22 | 11.8% | 32 | 10.8% | 54 | 11.2% |
| Application orientation | General tongue diagnosis / objectification | 119 | 63.6% | 163 | 55.3% | 282 | 58.5% |
|  | TCM syndrome / constitution assessment | 25 | 13.4% | 22 | 7.5% | 47 | 9.8% |
|  | Disease-specific application | 38 | 20.3% | 97 | 32.9% | 135 | 28.0% |
|  | General health assessment / prediction | 5 | 2.7% | 13 | 4.4% | 18 | 3.7% |
| AI / computational approach* | Conventional image processing / handcrafted features | 50 | 26.7% | 78 | 26.4% | 128 | 26.6% |
|  | Traditional machine learning | 34 | 18.2% | 47 | 15.9% | 81 | 16.8% |
|  | Deep learning / CNN | 78 | 41.7% | 155 | 52.5% | 233 | 48.3% |
|  | Transfer learning | 7 | 3.7% | 8 | 2.7% | 15 | 3.1% |
|  | Transformer / attention / LLM | 13 | 7.0% | 47 | 15.9% | 60 | 12.4% |
|  | Multimodal / feature fusion | 23 | 12.3% | 30 | 10.2% | 53 | 11.0% |
|  | Semi-/self-supervised or unsupervised learning | 13 | 7.0% | 13 | 4.4% | 26 | 5.4% |
|  | Explainable AI / uncertainty estimation | 1 | 0.5% | 11 | 3.7% | 12 | 2.5% |
|  | Other / unspecified AI-computational approach | 34 | 18.2% | 25 | 8.5% | 59 | 12.2% |
| Tongue-diagnosis task* | Image acquisition / calibration / quality control | 13 | 7.0% | 10 | 3.4% | 23 | 4.8% |
|  | Tongue segmentation / localization | 27 | 14.4% | 80 | 27.1% | 107 | 22.2% |
|  | Feature extraction / quantitative analysis | 86 | 46.0% | 108 | 36.6% | 194 | 40.2% |
|  | Tongue phenotype recognition / classification | 69 | 36.9% | 129 | 43.7% | 198 | 41.1% |
|  | TCM syndrome / constitution differentiation | 39 | 20.9% | 31 | 10.5% | 70 | 14.5% |
|  | Disease diagnosis / prediction / risk assessment | 43 | 23.0% | 110 | 37.3% | 153 | 31.7% |
|  | Multimodal / integrated diagnosis | 26 | 13.9% | 30 | 10.2% | 56 | 11.6% |
|  | Device / system / platform development | 24 | 12.8% | 65 | 22.0% | 89 | 18.5% |
|  | Review / bibliometric synthesis | 34 | 18.2% | 2 | 0.7% | 36 | 7.5% |
|  | Other / unspecified tongue-analysis task | 7 | 3.7% | 10 | 3.4% | 17 | 3.5% |
| Disease/application area* | Diabetes / metabolic disorders | 9 | 4.8% | 34 | 11.5% | 43 | 8.9% |
|  | Gastrointestinal / hepatobiliary disorders | 10 | 5.3% | 21 | 7.1% | 31 | 6.4% |
|  | Cancer / oncology | 14 | 7.5% | 24 | 8.1% | 38 | 7.9% |
|  | Cardiovascular / cerebrovascular disorders | 5 | 2.7% | 16 | 5.4% | 21 | 4.4% |
|  | Respiratory / infectious diseases | 4 | 2.1% | 7 | 2.4% | 11 | 2.3% |
|  | Renal / urologic disorders | 3 | 1.6% | 4 | 1.4% | 7 | 1.5% |
|  | Dermatologic / inflammatory disorders | 1 | 0.5% | 2 | 0.7% | 3 | 0.6% |
|  | Neurologic / psychiatric disorders | 1 | 0.5% | 5 | 1.7% | 6 | 1.2% |
|  | Other specified diseases | 3 | 1.6% | 14 | 4.7% | 17 | 3.5% |

**Note:** CNKI, n = 187; WoSCC, n = 295; total, n = 482. Application orientation, AI/computational approach, tongue-diagnosis task, and disease/application area were coded from titles and author keywords, with abstracts reviewed when necessary. Sections marked with an asterisk (*) permitted multiple classifications; therefore, percentages may exceed 100%. CNKI, China National Knowledge Infrastructure; WoSCC, Web of Science Core Collection; AI, artificial intelligence; TCM, Traditional Chinese Medicine.**Database-indexed document type and content-based classification were treated separately; therefore, publications coded as “review/bibliometric synthesis” were not necessarily indexed as Review Articles by the source database**

**Supplementary Table S3. Original Chinese bibliographic information for the top 10 most cited CNKI-indexed publications presented in Table 5**

| **Rank** | **First author** | **Year** | **Title** | **Journal** | **Total citations** | **Citations per year** |
| --- | --- | --- | --- | --- | --- | --- |
| 1 | 刘梦 | 2019 | 基于深度学习与迁移学习的中医舌象提取识别研究 | 中医杂志 | **119** | **17.00** |
| 2 | 李丹溪 | 2017 | 舌诊仪的发展及其在舌诊客观化研究中的应用现状 | 世界中医药 | **83** | **9.22** |
| 3 | 李渊彤 | 2020 | 基于深度学习的舌象特征分析 | 计算机科学 | **74** | **12.33** |
| 4 | 齐真 | 2015 | 基于数字图像处理技术的舌诊客观化临床应用进展 | 中华中医药杂志 | **66** | **6.00** |
| 5 | 江智泉 | 2022 | 人工智能背景下的中医舌诊客观化研究概述 | 计算机时代 | **63** | **15.75** |
| 6 | 阚红星 | 2016 | 一种2型糖尿病中医证型的舌图像识别方法 | 中国生物医学工程学报 | **63** | **6.30** |
| 7 | 卢运西 | 2021 | 中医舌象分割技术研究进展：方法、性能与展望 | 自动化学报 | **58** | **11.60** |
| 8 | 潘思行 | 2020 | 基于神经网络和支持向量机的中医体质辨识模型研究 | 世界科学技术-中医药现代化 | **57** | **9.50** |
| 9 | 樊威 | 2019 | 人工智能在中医舌诊中的应用探讨 | 光明中医 | **55** | **7.86** |
| 10 | 马龙祥 | 2020 | 基于高分辨率特征的舌象分割算法研究 | 计算机工程 | **52** | **8.67** |

**Note:** The original Chinese author names, article titles, and journal names are provided here; English translations are presented in Table 5 of the main manuscript.

**Supplementary Table S4. Characteristics of all keyword clusters identified in the CNKI and WoSCC datasets**

| Dataset | Cluster ID | Original cluster label | English translation | Size | Silhouette | Mean year | **Representative terms** |  |
| --- | --- | --- | --- | --- | --- | --- | --- | --- |
| CNKI | #0 | 图像分割 | Image segmentation | 29 | 0.994 | 2019 | 图像分割; 分类;中医舌诊;裂纹舌;颜色特征 |  |
| CNKI | #1 | 中医诊断 | TCM diagnosis | 28 | 0.878 | 2019 | 中医诊断; 图像处理;痤疮; 高光; 中医 |  |
| CNKI | #2 | 机器学习 | Machine learning | 26 | 0.984 | 2021 | 机器学习; 糖尿病; 2型糖尿病; 中西医; 预测模型 |  |
| CNKI | #3 | 中医 | Traditional Chinese medicine | 25 | 0.944 | 2021 | 特征融合; 图像识别; 辅助诊疗; 人工智能;中医 |  |
| CNKI | #4 | 客观化 | Objectification | 24 | 0.799 | 2021 | 中医辨证; 应用研究; 研究进展; 结直肠癌;智能诊疗 |  |
| CNKI | #5 | 舌诊 | Tongue diagnosis | 23 | 0.979 | 2020 | 图像处理;数字图像处理技术; 异常舌;目标检测; 糖尿病 |  |
| CNKI | #6 | 舌苔 | Tongue coating | 13 | 0.929 | 2020 | 舌苔;舌体分割;舌象分析; 中医舌诊; 深度学习 |  |
| CNKI | #7 | 神经网络 | Neural network | 9 | 0.967 | 2022 | 甲状腺功能亢进; 形体特征; 用药规律; tabnet;国医大师 |  |
| CNKI | #8 | 深度学习 | Deep learning | 9 | 1 | 2022 | 中医体质;机器视觉; 深度学习;中医; 舌色 |  |
| CNKI | #9 | 智能舌诊 | Intelligent tongue diagnosis | 8 | 1 | 2022 | 智能舌诊; 智能化诊断;条纹池化;舌像分割;轻量化 |  |
| CNKI | #10 | 亚健康 | Subhealth | 6 | 0.993 | 2022 | 亚健康;肾阳虚证;脾阳虚证; 图像特征;舌诊 |  |
| CNKI | #12 | 持续学习 | Continuous learning | 4 | 1 | 2023 | 中医舌色苔色分类;全局-局部混合注意力机制;协同分类;持续学习; 机器视觉;深度学习 |  |
| WoSCC | #0 | MobileNetV2 architecture | — | 34 | 0.853 | 2021 | deep learning; tongue image; artificial intelligence; tongue diagnosis; syndrome differentiation |  |
| WoSCC | #1 | Deep learning | — | 34 | 0.972 | 2020 | deep learning; tongue image; tongue diagnosis; artificial intelligence; syndrome differentiation |  |
| WoSCC | #2 | Tongue features | — | 33 | 0.861 | 2022 | tongue features; objective tongue diagnosis; traditional chinese medicine syndrome; clustering analysis; diabetic kidney disease |  |
| WoSCC | #3 | Machine learning | — | 32 | 0.906 | 2019 | machine learning; tongue diagnosis; traditional medicine; ethnic differences; image processing |  |
| WoSCC | #4 | Convolutional neural network | — | 30 | 0.917 | 2021 | convolutional neural network; traditional chinese medicine; tongue segmentation; supervised learning; loss function |  |
| WoSCC | #5 | Tongue segmentation | — | 27 | 0.949 | 2020 | tongue segmentation; encoder-decoder network; separable convolution; global refinement; semisupervised learning |  |
| WoSCC | #6 | Diabetes mellitus | — | 24 | 0.952 | 2018 | tongue diagnosis; feature extraction; medical diagnostic imaging; diabetic tongue image; confidence estimation |  |
| WoSCC | #7 | Facial image | — | 23 | 0.92 | 2019 | tongue image; facial image; fatty liver; collaborative representation; discriminative representation |  |
| WoSCC | #8 | Artificial intelligence | — | 20 | 0.964 | 2022 | artificial intelligence; traditional chinese medicine; tongue images; tongue coating microbiome; gastric cancer |  |
| WoSCC | #9 | Computational modelling | — | 14 | 0.958 | 2020 | visible hyperspectral image system; cielab values; image colour analysis; medical image processing; approximate spectral range |  |
| WoSCC | #10 | Acupuncture | — | 11 | 0.936 | 2018 | non-malignant upper gastrointestinal disorders; tongue examination; traditional chinese medicine; evidence-based medicine; heart rate |  |
| WoSCC | #11 | Attention mechanism | — | 8 | 1 | 2021 | attention mechanism; convolutional networks; regional detailed features; tongue image modeling; disease-position recognition |  |
| WoSCC | #12 | Health information | — | 6 | 0.989 | 2023 | chinese medicine constitution classification; multimodal deep learning; tongue image; face image; pulse wave; health information |  |

**Note:** Cluster labels were generated using the log-likelihood ratio (LLR) method in CiteSpace. Size indicates the number of keyword nodes within each cluster; silhouette reflects within-cluster consistency; and mean year indicates the average publication year of the cluster. Original Chinese labels and English translations are both provided for CNKI clusters. CNKI, China National Knowledge Infrastructure; WoSCC, Web of Science Core Collection.Cluster IDs follow the original CiteSpace output. Cluster #11 was not present in the final CNKI clustering output, and the clusters were not manually renumbered.

**Supplementary Table S5. Original Chinese keywords and standardized English translations used in keyword burst analysis**

| **No.** | **Original Chinese keyword** | **Standardized English translation** | **Burst period** | **Strength** |
| --- | --- | --- | --- | --- |
| 1 | 图像处理 | Image processing | 2015–2016 | 1.49 |
| 2 | 质量评价 | Quality evaluation | 2015–2017 | 1.12 |
| 3 | 图像识别 | Image recognition | 2015–2016 | 1.05 |
| 4 | 图像分割 | Image segmentation | 2016–2017 | 2.69 |
| 5 | 舌苔 | Tongue coating | 2016–2018 | 1.03 |
| 6 | 舌象识别 | Tongue recognition | 2018–2019 | 1.72 |
| 7 | 面诊 | Facial diagnosis | 2019–2020 | 1.24 |
| 8 | 舌象 | Tongue image / tongue appearance | 2019–2021 | 1.17 |
| 9 | 大数据 | Big data | 2019–2020 | 1.11 |
| 10 | 迁移学习 | Transfer learning | 2020–2021 | 2.61 |
| 11 | 舌体分割 | Tongue segmentation | 2020–2021 | 1.56 |
| 12 | 研究进展 | Research progress | 2021–2023 | 1.17 |
| 13 | 特征分析 | Feature analysis | 2021–2023 | 1.17 |
| 14 | 舌象分类 | Tongue image classification | 2022–2023 | 1.47 |
| 15 | 深度学习 | Deep learning | 2023–2024 | 2.95 |

**Footnote:**Chinese keywords were translated into English using standardized terminology related to artificial intelligence, medical imaging, and traditional Chinese medicine. The original Chinese terms were retained to ensure traceability and avoid ambiguity during cross-language interpretation.

**Supplementary Table S6. The BIBLIO checklist for reporting the bibliometric reviews of the biomedical literature**

| **Section/Topic** | **Item No.** | **Checklist item** | **Reported on page No.** |
| --- | --- | --- | --- |
| **Title** |  |  |  |
| Identification | 1 | Identify the report as a bibliometric review in the title. | 1 |
| Issues/topics | 2 | Indicate the key issues/topics under investigation and coverage of time period. | 1 |
| **Abstract** |  |  |  |
| Structured summary | 3 | Structured summary including (as applicable): background, methods, results (key findings) and conclusions. | 2 |
| **Introduction/ Background** |  |  |  |
| Justification/ Rationale/ Explanation | 4 | Present review of existing knowledge and epidemiological information. | 3-5 |
| Objectives | 5 | Statement of the objective (s) or question (s). | 5-6 |
| **Methods** |  |  |  |
| Search engines (data sources) | 6 | Describe all information sources (such as electronic databases, contact with study authors, trial registers or other grey literature sources). | 6 |
| Search strategy | 7 | Keywords and systematization criteria (date of search, language, type of document) for the search. | **7–8; Supplementary Table S1** |
| Time period | 8 | The period that the review covers and the justification. | 6 |
| Eligibility criteria | 9 | Describe all inclusion and exclusion criteria; languages; study design, type of publication and time period. | 8-9 |
| Data refinement (data selection procedure) | 10 | Remove the irrelevant articles; inspection to eliminate duplicate and unrelated articles (after evaluation of the title, abstract and content). | 9-10 |
| Quality assessment (optional) | 11 | Assessment of papers by three authors and the use of assessing checklists. | **N/A (optional; rationale on p.11)** |
| Data synthesis | 12 | Describe the methods used for summarizing, handling, synthesis, tabulations or schematic displays. Describe how the data were analysed. | 10-12 |
| **Results** |  |  |  |
| Descriptive findings (statistics) | 13 | - Provide details of the search and selection process in a flow diagram.  - Number of citations retrieved (number of publication, year of publication, type of documents, country of publication, articles with the highest impact, most impactful authors, most impactful articles, authors with the highest production, top journals, top institutions, …) | **9, 12–16; Figure 1; Tables 1–6** |
| Schematic map and trend | 14 | Summarize and/or present the schematic maps and trends using an appropriate software to present citations, journals, authors, top journals, time trends, emerging literature, and any relevant indicators (as applicable) [1-5]. | **12–20; Figures 2–10** |
| Tabulation and summarizing the findings | 15 | General recommendation: Studies under consideration could be summarized and organized by different subtitles and different scenarios. Regardless, results need to be presented in separate tables covering each subtitle. The followings are some options that could help to summarize the findings.  *Option 1:*  - Start the presentation with a historical view [when and who first published on the topic].  - Report on review papers. The result should be listed in a separate table. Also, specify the review type (scoping review, narrative review, systematic review, and meta-analysis).  - Summarize the findings according to the study designs and main study types.  *Option 2:*  - Start the presentation with a historical view [when and who first published on the topic].  - Report on review papers. The result should be listed in a separate table. Also, indicate the review type (scoping review, narrative review, systematic review, and meta-analysis) should be specified.  - Summarize the findings according to outcome measures or populations. For example, see [6].  *Option 3:*  - Start the presentation with a historical view [when and who first published on the topic].  - Report on review papers. The result should be listed in a separate table. Also, specify the review type (scoping review, narrative review, systematic review, and meta-analysis).  - Summarize the findings according to concept [7].  *Option 4.*  - Start the presentation with a historical view [when and who first published on the topic].  - Report on review papers. The result should be listed in a separate table, and also specify the review type (scoping review, narrative review, systematic review, and meta-analysis).  - Summarize the findings according to different subtitles relevant to the main topic [8]. | **12–13; Supplementary Table S2** |
| Synthesis of findings | 16 | Synthesize the findings as much as possible, find the gap, and propose a model, hypothesis, etc. (if applicable). | 20-27 |
| **Discussion** |  |  |  |
| Summary of evidence | 17 | Summarize the main findings. The findings should be presented in more "general" or "accessible" terms. | 20-21 |
| Interpretation | 18 | Include interpretation consistent with results. Explanations for observed outcomes, similarities, and differences reported would be essential. | 20-25 |
| Strengths and limitations | 19 | Discuss the strengths and limitations. | 25-27 |
| Conclusion(s) | 20 | Provide a general interpretation of the results with respect to the review questions and objectives, as well as potential implications. | 26-27 |
| 1. McDougal L, Dehingia N, Cheung WW, Dixit A, Raj A. COVID-19 burden, author affiliation and women's well-being: A bibliometric analysis of COVID-19 related publications including focus on low-and middle-income countries. eClinicalMedicine 2022; 52: 101606.  2. Henstock L, Wong R, Tsuchiya A, Spencer A. Behavioral theories that have influenced the way health state preferences are elicited and interpreted: A bibliometric mapping analysis of the ttime trade-off method with VOSviewer visualization. Front Health Serv 2022; 2: 848087.  3. Bodea F, Bungau SG, Negru AP, Radu A, Tarce AG, Tit DM, et al. Exploring new therapeutic avenues for ophthalmic disorders: Glaucoma-related molecular docking evaluation and bibliometric analysis for improved management of ocular diseases. Bioengineering 2023; 10(8): 983.  4. Sang XZ, Wang CQ, Chen W, Rong H, Hou LJ. An exhaustive analysis of post-traumatic brain injury dementia using bibliometric methodologies. Front Neurol 2023; 14: 1165059.  5. Ramli MI, Hamzaid NA, Engkasan JP, Usman J. Respiratory muscle training: a bibliometric analysis of 60 years’ multidisciplinary journey. Biomed Eng Online 2023; 22(1): 50.  6. Akosman I, Kumar N, Mortenson R, Lans A, De La Garza Ramos R, Eleswarapu A,et al. Racial differences in perioperative complications, readmissions, and mortalities after elective spine surgery in the United States: A systematic review using AI-assisted bibliometric analysis. Glob Spine J 2023: 21925682231186759.  7. Tavousi M, Mohammadi S, Sadighi J, Zarei F, Kermani RM, Rostami R, Montazeri A. Measuring health literacy: A systematic review and bibliometric analysis of instruments from 1993 to 2021. Plos One 2022; 17(7): e0271524.  8. Montazeri A. Health-related quality of life in breast cancer patients: A bibliographic review of the literature from 1974 to 2007. J Exp Clin Cancer Res 2008; 27: 32. | | | |

**Rights and permissions:** The original source of the checklist is: Montazeri A, Mohammadi S, M.Hesari P, Ghaemi M, Riazi H, Sheikhi‑Mobarakeh Z. Preliminary guideline for reporting bibliometric reviews of the biomedical literature (BIBLIO): a minimum requirements. *Systematic Reviews* 2023; 12: 239. doi.org/10.1186/s13643-023-02410-2 The article is licensed under a Creative Commons Attribution 4.0 International License (<http://creativecommons.org/licenses/by/4.0/>). A changes was made to the original checklist to add in full references to the cited sources.
